# Explainable machine learning framework to predict personalized physiological aging

**DOI:** 10.1111/acel.13872

**Published:** 2023-06-10

**Authors:** David Bernard, Emmanuel Doumard, Isabelle Ader, Philippe Kemoun, Jean‐Christophe Pagès, Anne Galinier, Sylvain Cussat‐Blanc, Felix Furger, Luigi Ferrucci, Julien Aligon, Cyrille Delpierre, Luc Pénicaud, Paul Monsarrat, Louis Casteilla

**Affiliations:** ^1^ RESTORE Research Center Université de Toulouse, INSERM 1301, CNRS 5070, EFS, ENVT France; ^2^ Université Toulouse 1 – Capitole, Institute of Research in Informatics (IRIT) of Toulouse, CNRS Toulouse France; ^3^ Oral Medicine Department and Hospital of Toulouse Toulouse Institute of Oral Medicine and Science, CHU de Toulouse Toulouse France; ^4^ UFR Santé, Département Médecine, Institut Fédératif de Biologie, CHU de Toulouse Toulouse France; ^5^ Artificial and Natural Intelligence Toulouse Institute ANITI Toulouse France; ^6^ Biomedical Research Centre, National Institute on Aging NIH Baltimore Maryland USA; ^7^ CERPOP, UMR1295 (Equity) Université P. Sabatier Toulouse France

**Keywords:** artificial intelligence, biological age, Explainability, healthy aging, machine learning, personalized medicine, physiological age, Rejuvenative therapy

## Abstract

Attaining personalized healthy aging requires accurate monitoring of physiological changes and identifying subclinical markers that predict accelerated or delayed aging. Classic biostatistical methods most rely on supervised variables to estimate physiological aging and do not capture the full complexity of inter‐parameter interactions. Machine learning (ML) is promising, but its black box nature eludes direct understanding, substantially limiting physician confidence and clinical usage. Using a broad population dataset from the National Health and Nutrition Examination Survey (NHANES) study including routine biological variables and after selection of XGBoost as the most appropriate algorithm, we created an innovative explainable ML framework to determine a Personalized physiological age (PPA). PPA predicted both chronic disease and mortality independently of chronological age. Twenty‐six variables were sufficient to predict PPA. Using SHapley Additive exPlanations (SHAP), we implemented a precise quantitative associated metric for each variable explaining physiological (i.e., accelerated or delayed) deviations from age‐specific normative data. Among the variables, glycated hemoglobin (HbA1c) displays a major relative weight in the estimation of PPA. Finally, clustering profiles of identical contextualized explanations reveal different aging trajectories opening opportunities to specific clinical follow‐up. These data show that PPA is a robust, quantitative and explainable ML‐based metric that monitors personalized health status. Our approach also provides a complete framework applicable to different datasets or variables, allowing precision physiological age estimation.

## Graphical abstract

Personalized physiological age (PPA) is a robust, quantitative and explainable machine learning‐based metric that monitors health status. Our approach provides a complete framework applicable to different datasets or variables, allowing precision physiological age estimation.

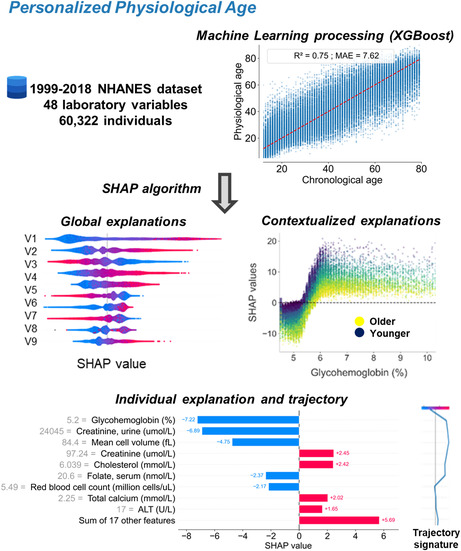

**Abbreviations**
AIC: Akaike Information Criterion
ALT: Alanine amino Transferase
AST: Aspartate Transferase
BUN: Blood Urea Nitrogen
DL: Deep Learning
GGT: Gamma‐Glutamyl Transferase
HbA1c: Glycated hemoglobin
HD: Homeostatic Dysregulation
KDM: Klemera‐Doubal
MAE: Mean Absolute Error
MCV: Mean Cell Volume
ML: machine learning
MLP: Multi‐Layer Perceptron
NHANES: National Health and Nutrition Examination Survey
PDP: Partial Dependences Plot
PPA: Personalized physiological age
RFE: Recursive Feature Elimination
RNN: Neural networks
SHAP: SHapley Additive exPlanations
UMAP: Uniform Manifold Approximation and Projection

## 1 INTRODUCTION

The expansion in the aging population and concomitant age‐associated increase in chronic diseases and disabilities have resulted in a rising global socio‐economic burden. As a consequence in recent years, the main goal of aging research tends to develop approaches promoting healthy aging and preventing loss of autonomy. In this regard, the WHO identified intrinsic capacity and associated functions as a major target to facilitate accurate monitoring of healthy aging (Beard et al., 2016). At the same time, the geroscience paradigm emphasized changes in physiological biology along aging as the primary cause for chronic disease (Kennedy et al., 2014). Of note, geroscience investigations are often conducted at the cellular or molecular scales, far from the integrative view required to address clinical aspects of healthy aging. Indeed, aging results from multifactorial small deviations in interdependent physiological processes starting at earliest age and leading to highly variable health trajectories when comparing people of the same chronological age (Li et al., 2015). We recently proposed that healthy aging and geroscience could be reconciled through the gerophysiology perspective (Ferrucci et al., 2020; Kemoun et al., 2022).

Several studies have proposed operational definitions of a “biological age.” These include sets of molecular biomarkers, such as the epigenetic clock or epigenetic markers (Hägg et al., 2019; Horvath & Raj, 2018; McCrory et al., 2021), or alternative integrative strategies (Klemera‐Doubal, i.e., KDM, Levine methods, homeostatic dysregulations, allostatic load) that are based on linear or non‐linear combinations of phenotypic measures of aging (Cohen et al., 2013; Karlamangla et al., 2002; Klemera & Doubal, 2006; Liu et al., 2018). In most cases, individual biological age is estimated by comparing a set of variables from referent populations. For example, the homeostatic dysregulation estimates the Mahalanobis distance deviation of biomarkers from a referent population considered to have optimal functions (Cohen et al., 2013; Liu et al., 2018) while KDM (Klemera & Doubal, 2006) estimates the relative age‐dependent deviation from a referent population with different ages defined from linear regressions of several biomarkers. All of these measures appear to be correlated with each other (Hastings et al., 2019; McCrory et al., 2019, 2020). In all cases, most strategies remained hypothesis‐driven, used a limited number of pre‐selected variables (socio‐economic, clinical, and/or biological variables) and hence share the risk of ignoring important variables in biological aging (Klemera & Doubal, 2006).

The recent rising development of machine learning (ML) has revolutionized data mining by exploiting databases. In particular, ML strategies appear perfectly suited for studying integrated phenotypes in large and high‐dimensional databases. ML computes many covariates even in complex interactions, regardless of their yields and nature, may outperforming common statistical approaches (Bi et al., 2019; Shin et al., 2021). In the field of biological age estimation, a wide variety of data may be integrated, from images (brain magnetic resonance imaging, chest radiology, retinal or face photography; Lombardi et al., 2021; Nusinovici et al., 2022; Raghu et al., 2021), to physical activity data, up to blood biomarkers, gut microbiome or genomic data (Galkin et al., 2020, 2021). Deep learning (DL) has offered new powerful ways of handling some of these data types, in particular images and sequential data like physical activity. Convolutional neural networks (CNN) and recurrent neural networks (RNN) appear particularly relevant for knowledge extraction from images (Cole et al., 2017) and sequential data (Rahman & Adjeroh, 2019) for biological age estimation, respectively. Considering the adequacy of ML models with tabular data, a DL framework based on a deep neural network hybridized with an ElasticNet model has been proposed for biological age estimation based on blood chemistry (Mamoshina et al., 2018; Putin et al., 2016), revealing the existence of population‐specific aging patterns. Unfortunately, DL models displayed little explainability (Cohen et al., 2016; Putin et al., 2016), a feature favoring medical professional acceptance and use, and relevant to provide potential explanatory pathophysiological hypotheses (Amann et al., 2020).

Using the National Health and Nutrition Examination Survey (NHANES) database gathering routine laboratory values for physiological functions, we built a comprehensive analytic ML‐based strategy to (i) define and estimate physiological age and capture differences to chronological age that show accelerated or delayed aging, (ii) provide the relative weights of the variables in comparison with an average individual in the total population or in comparison with an individual sharing chronological age (global and contextualized explainability, respectively), (iii) unravel how variations of each variable quantitatively affect the explanations, (iv) provide a range of biological values associated with healthy aging, and (v) identify different aging trajectories by clustering profiles of identical contextualized explanations.

## 2 MATERIALS AND METHODS

### 2.1 Data source

All data from the NHANES study were collected at the Centers for Disease Control and Prevention website [https://wwwn.cdc.gov/Nchs/Nhanes], from NHANES 1999–2000 to NHANES 2017–2018. Data were available from multiple files, each containing a set of variables for a specific year. All these files were merged to obtain a single database containing all the available data for each subject (with the SEQN—the id of the subject—providing the joint between all the information). This approach allowed the generation of a unique database that gathers all the subjects examined, as well as related data, over a 20‐year span.

### 2.2 Inclusion criteria of variables for algorithm development

In the database, only variables from laboratory parameters were selected, with the exception of pollutants, toxic exposure, and infection‐related variables. We chose simple biological variables routinely assessed for diagnosis and treatment‐monitoring because: (i) these variables span the landscape of physiological homeostasis and their variations have been widely documented, and (ii) their routine use makes personalized physiological age (PPA) easy to translate into clinical settings or studies, including longitudinal analyses.

Chronological age and gender were taken from demographic data. In addition, the age limit of 79 years was chosen because depending on the time period, 1999–2006 or 2007–2018, NHANES defined two different ages, 85 or 80 years, respectively, as cutoff ages for their coding. Participants under 12 years old were excluded from the database because many laboratory variables were only collected in this age group.

### 2.3 Variable selection and merging

To generate a consistent and large database, with a maximal number of common biological variables for subjects, we performed a manual data cleaning to eliminate redundant outcomes, both within the same year and in different years (Supplementary Text). After this step, and considering the distribution of the number of available variables for a given number of subjects, the largest dataset with the minimum amount of missing data was defined. The cutoff for this distribution selected variables with at least 50,000 individuals. Individuals with more than 10% missing values were also dropped from database. After processing, the selected dataset contained 60,322 individuals with 48 laboratory variables (Table S1) and limited missing data (0.6% of data, Figure S2).

### 2.4 Machine learning processing

#### 2.4.1 Handling missing data

Processing for data visualization and Machine Learning is described below (except for XGBoost which is able to manage missing data natively). A multivariate single imputation method for missing data based on an iterative imputer was implemented, using a Bayesian Ridge model as the estimator at each step of the round‐robin imputation (van Buuren & Groothuis‐Oudshoorn, 2011).

#### 2.4.2 Data visualization

A projection was made using the Uniform Manifold Approximation and Projection (UMAP) algorithm (McInnes et al., 2018). The method builds an undirected graph using K‐Nearest‐Neighbors on the entire dataset, viewed in a 2D scatter plot.

#### 2.4.3 Development of the machine learning pipeline

The dataset was divided into a training and a test set in an 80:20 proportion (Panesar, 2021) to train machine learning algorithm per se with chronological age as a target value. GrootCV algorithm was implemented using the “*arfs 0.2.3*” package to eliminate variables that did not contribute to the estimation of chronological age (cross‐validated feature selection based on lightGBM and feature importance derived from SHAP importance).

#### 2.4.4 Model robustness assessment

Three classes of models were compared: (i) Tree‐based with Decision Tree, Random Forests (“*scikit‐learn 1.0.1*” package) and Gradient Boosting Machine XGBoost (“*xgboost 1.5.1*” package), (ii) Neural Networks with Multi‐Layer Perceptron (MLP), and (iii) penalized linear models with Elastic Net through the *“scikit‐learn 1.0.1”* package. Grid‐search exploration of hyperparameters with cross‐validation was performed on training dataset for each model using the “*optuna 2.10.0*” and “*ray 1.9.1*” packages (list of the hyperparameters grid search in Table S2). Model training aimed at minimizing the mean absolute error (MAE). Models were evaluated based on their results on the train and test set in terms of *R* ^2^ (coefficient of determination) and MAE. Standard deviations were provided for the train set, using fivefold cross‐validation. To avoid performance discrepancy across the age group during model training, a custom objective function was introduced for XGBoost. It used a normalization per chronological age to correct the gradient used by the model and thus correct its error at the next iteration (Equation 1).

(1) $$\mathrm{gra}d_{i}=\left({\hat{y}}_{i}-y_{i}\right)\times \left|\frac{\frac{\sum_{j\in \mathrm{age}\left(i\right)}\left({\hat{y}}_{j}-y_{j}\right)}{\left|\mathrm{age}\left(i\right)\right|}}{\frac{\sum_{k=1}^{N}\left({\hat{y}}_{k}-y_{k}\right)}{N}}\right|$$

where $\mathrm{gra}d_{i}$ is the gradient to be calculated for the *i*th individual, $\hat{y}$ is the prediction of the model for a given iteration, y is the chronological age, $\mathrm{age}\left(i\right)$ represents all individuals that display the same age as the *i*th individual, and N is the total number of individuals.

#### 2.4.5 Model explainability

SHapley Additive exPlanations (SHAP) was used as explainability model, “*shap 0.39.0*” package, to give local explanations and allow computation of the contribution of each variable to the prediction for each individual (Lundberg & Lee, 2017). Shapley values originate from the cooperative game theory field and are the average marginal contribution of a variable among all possible coalitions. The SHAP value for each variable for an individual corresponds to the contribution in age (positive or negative) of this variable to the age prediction of this individual. The predicted age of an individual PPA is therefore the sum of the total contributions of these variables to the base value (the mean predicted age of the individuals of the dataset).

#### 2.4.6 Contextualized explainability

To define the influence of each laboratory variable of the model for an individual, a separate explanation model was trained for each age group, thus defining a contextualized explanation. The contextualized SHAP value of a variable for a given individual corresponded to the contribution in age (positive or negative) of this variable, not relative to the whole population, but relative to other individuals sharing the same chronological age (with the mean predicted age of the individuals sharing the same chronological age as the base value). Summing variables gave the individual PPA deviation from chronological age relative to the population that shares the same chronological age.

#### 2.4.7 Partial dependence computations

Partial dependences for each variable were computed and plotted (PDP). PDP represents the contextualized SHAP contribution of a variable according to its raw value. An example of interpretation is given in Figure S3.

### 2.5 Model validation and robustness

#### 2.5.1 Recursive feature elimination for obtaining a reduced model

To reduce the number of variables without degrading the quality of the model, the principle of the recursive feature elimination (RFE) algorithm was followed (Guyon et al., 2002). After hyperparameter tuning (as previously described), training the XGBoost model with custom loss and computing SHAP values, the variable with the least importance was removed from the dataset. These steps were repeated until all variables were removed and the evolution of the *R* ^2^ metric was monitored. The number of features necessary to obtain a model with less than 1% decrease of *R* ^2^ compared to a complete model was considered as a performant reduced model.

#### 2.5.2 Survival analysis

The validation of the physiological age model was performed using mortality data from the 2015 public mortality files (Lu et al., 2021) merged with the NHANES database based on the SEQN of the respondents for years 1999–2015. The hypothesis tested was that a higher PPA deviation may be predictive of an increased risk of mortality, and conversely, a lower PPA predicts decreased risk. PPA was also compared to homeostatic dysregulation (HD) and KDM metrics using the same set of variables as PPA to compute them, following the methodology previously described (Hastings et al., 2019). A multivariate Cox proportional hazards regression model was then performed to compute the mortality hazard ratio of the PPA deviation (categorized into deciles) after adjustment on the chronological age and NHANES year of subject inclusion.

#### 2.5.3 Validation on demographic, questionnaire and examination data

The hypothesis tested was that a higher PPA deviation reflected the overall poor, degraded health known to be the case in socially vulnerable and/or clinically at risk populations. We then assumed that PPA deviation was higher in these populations. PPA was compared to HD and KDM. The following socio‐demographic variables were considered: family poverty index ratio, the family income, ethnicity, and education level. The following medical variables and categories were considered: body mass index BMI, tobacco consumption, alcohol consumption, sedentarity, systolic blood pressure, presence of chronic systemic diseases (digestive, cardiovascular, metabolic, eye, urogenital, respiratory tract, immune system, musculoskeletal diseases, and neoplasms), abdominal aortic calcification score AAC24 (Lewis et al., 2016), and drug counts. Details about each validation variable were presented in Table S3.

#### 2.5.4 Contextualized SHAP values clusterization

To unravel similar explainability profiles and identify healthy aging trajectories, contextualized SHAP values were clustered. An agglomerative clustering technique has been used, with ward algorithm and Euclidean distance for linkage. Clustering results were then visualized on UMAP. To define a unique cluster signature with variables (by the contextualized explainability) allowing discrimination of at least two clusters, a Mann and Whitney test was performed for each variable per pair of clusters together with the corresponding effect size *r*. Only variables with at least a medium effect (*r* ≥ 0.3) and a significant *p*‐value after adjustment with the Benjamini–Hochberg test false discovery rate (FDR) were retained. For each cluster, the mean of the contextualized SHAP values was computed to produce an explanation of the cluster. For each cluster, explanations were presented in decision plots showing, on average, each variable's contribution to PPA deviation.

## 3 RESULTS

### 3.1 Definition and estimation of physiological age, that also captures differences with chronological age

**FIGURE 1:**
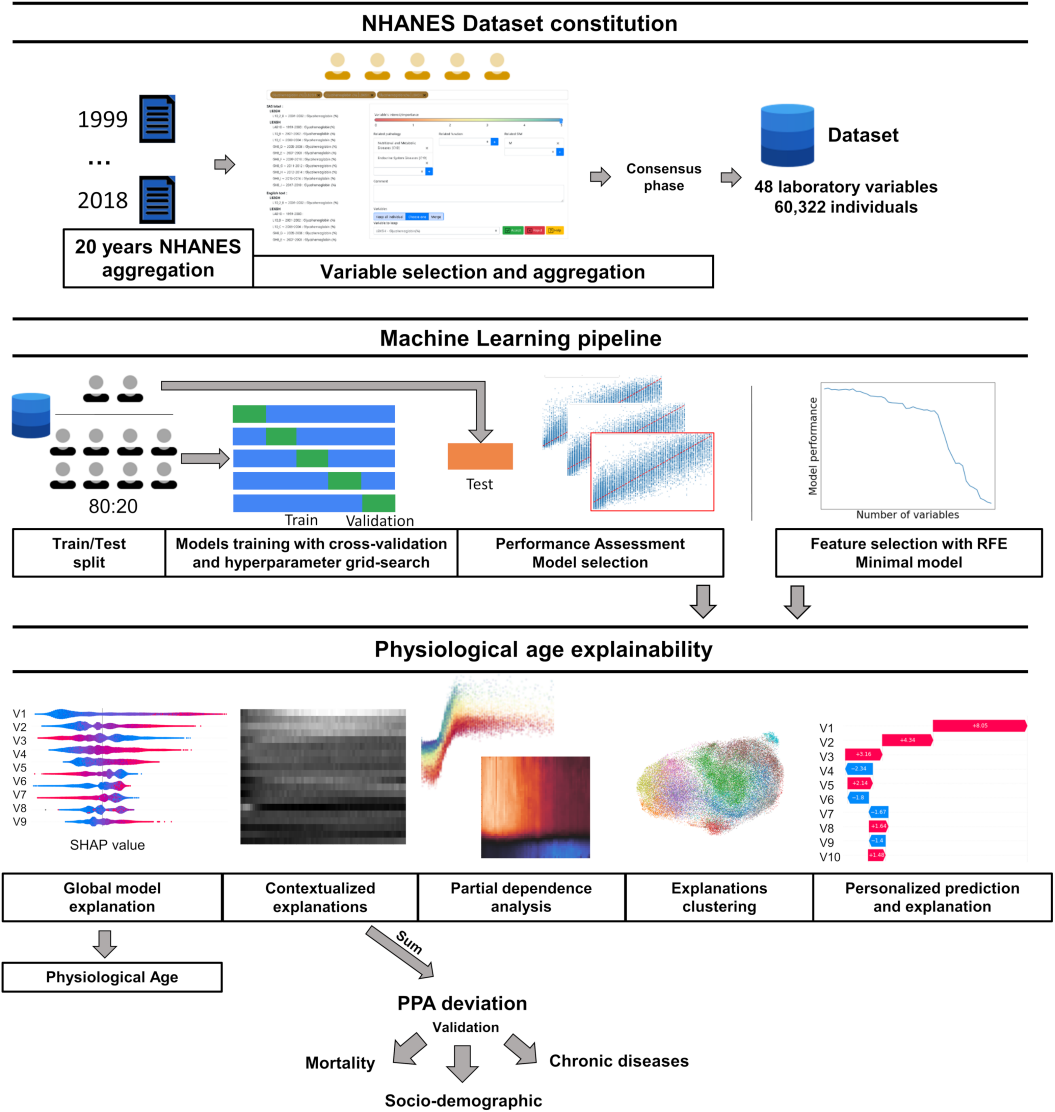
Machine learning analysis pipeline. All data from the National Health and Nutrition Examination Surveys (i.e., NHANES study) 1999–2018 were collected. A large, consistent database containing the maximal number of common biological variables reported on the maximal number of subjects, and with minimal missing data was generated. This resulted in a dataset with 60,402 individuals with 48 biological variables and 0.01% missing data. Using this dataset, five classes of algorithm models were trained, tested and compared based on performance. The XGBoost model with custom loss was considered (see Figure 2), and explainability was computed using SHAP values for the personalized physiological age (PPA) estimation. Deviation of PPA from chronological age is therefore the sum of the contextualized SHAP contributions of all the laboratory variables for a given subject (PPA deviation). Partial dependence plots and heatmaps of SHAP values also identify the precise range of biological values and thresholds for each variable and age group delineating accelerated or reduced aging. Clustering of SHAP values identifies specific PPA profiles. Finally, using recursive feature elimination, the list of variables was reduced to 26 biological variables without significant loss of model performance, providing a ready‐to‐use personalized and explainable model that is potentially clinically useful for monitoring physiological age to achieve healthy aging. PPA deviation was validated as a predictor of lifespan but also a risk factor for chronic diseases.

To develop an analytical framework unraveling personalized physiological age (PPA) from routine biological variables, we first merged and filtered data sources to obtain a clean, robust, and workable dataset. We next selected the best machine learning strategies including train/test splitting, feature selection, model selection, and optimization through performance comparison. We next developed a comprehensive explainability process to reveal a new metric for variables defining PPA. Finally, we validated the new metric on socio‐demographic data to predict mortality and chronic diseases (Figure 1).

#### 3.1.1 Building a comprehensive and robust dataset from the NHANES data

To define PPA using a state‐of‐the‐art and explainable ML‐based framework with common biological variables, we built the largest, most consistent and comprehensive dataset (Figure 1): (i) all NHANES data from 1999 to 2018 were merged, giving 36,945 variables, (ii) laboratory variables were selected and aggregated using a dedicated web interface (Figure S1), and (iii) the largest dataset fitting inclusion criteria with a minimal missing data was defined (Figure 1, Figure S2). Once variables with sufficient subjects and a low rate of missing data are filtered out, the final dataset included 48 laboratory variables (Table S1) for 60,322 individuals (30,747 females and 29,575 males, mean age 39.3 ± 19.7 and 39.5 ± 20.2 years, respectively). The distribution of individuals by age (Figure S2A) showed that the amount of data from 12 to 20 years was two times that of other ages, with a 25% decrease of available subjects from 70 to 79 years old. The different age groups showed no major gender imbalance. The amount of missing data was 0.1% of the total (linked to missing C‐reactive protein CRP, folate, albumin, and creatinine values) and uniformly distributed across age and sex (Figure S2b,c). An imputation method for missing data was implemented, except for XGBoost machine learning algorithm, which is able to manage missing data. In the 2D UMAP data visualization projection, the oldest subjects were predominantly clustered to the left and center of the UMAP, and clear gender symmetry was highlighted along a diagonal (Figure S4).

Altogether, we succeeded in building the largest possible dataset from the whole NHANES database corresponding to 60,322 individuals with very limited missing data.

#### 3.1.2 Selection of the best explainable algorithm to define PPA

**FIGURE 2:**
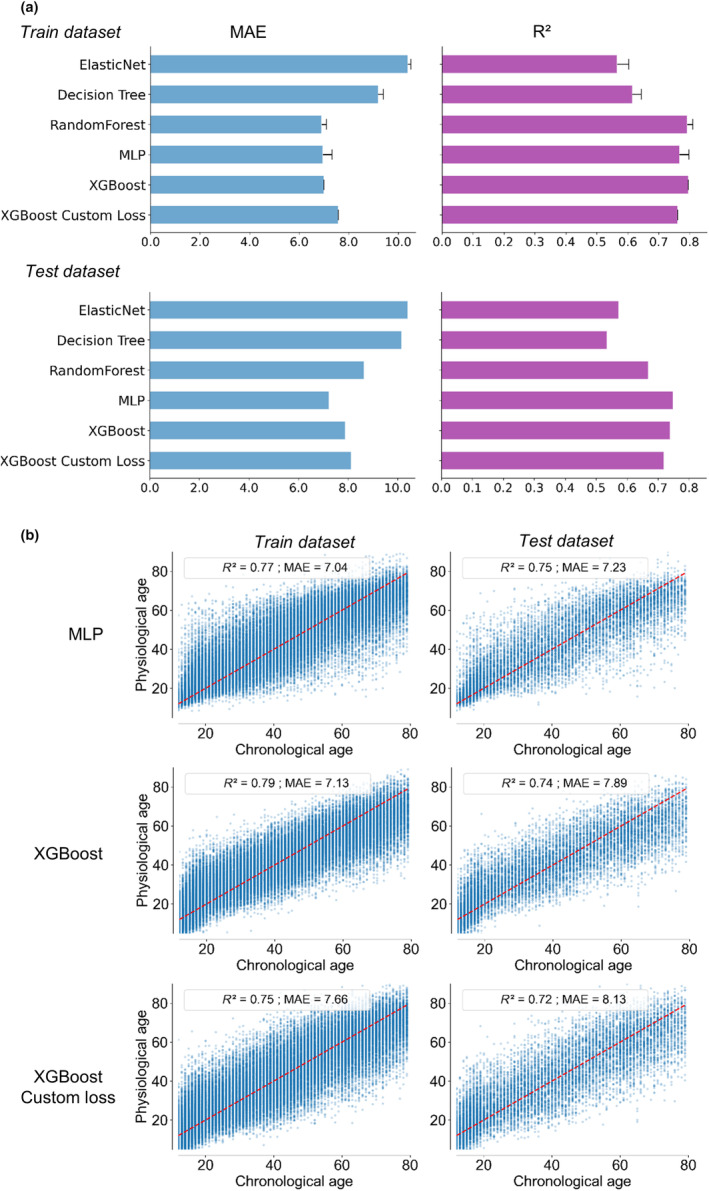
Model selection of different classes of machine learning models. (a) Several classes of models were tested to estimate physiological age, defined as the chronological age predicted by the model. An optimization of the hyperparameters of each model was performed on the training dataset, and the final achieved performance tested on the training and test datasets (coefficient of determination *R* ^2^ and mean absolute error MAE). MultiLayer Perceptron MLP and XGBoost model achieved the best performances. (b) Graphical representation of the predicted physiological age defined using MLP, XGBoost and XGBoost with Custom loss as function of chronological age. The red line highlights situations where physiological age is identical to chronological age. Custom loss applied to XGBoost improved XGBoost, by moderating the performance discrepancy across the age groups.

To test different machine learning algorithms, we split the dataset into training and test datasets (80% and 20%, respectively). No age and gender imbalance was found between train and test datasets (Figure S5). The number of variables was first reduced to 44, using GrootCV feature selection to remove variables with too little impact on the behavior of the ML model (Table S1). Three classes of machine learning algorithms were compared: tree‐based models (Decision Tree, Random Forests and XGBoost), a regularized regression method (ElasticNet, a method with both L1 and L2‐norm regularization of the coefficients), and a neural network (MultiLayer Perceptron, MLP). Using the training dataset, a grid‐search exploration of hyperparameters with a fivefold cross‐validation was performed for each model (Table S2). Comparing *R* ^2^ and MAE on test dataset, XGBoost and MLP performed the best, with similar performances and the lowest standard deviations during cross‐validation for XGBoost (Figure 2a,b; Figure S6). Given the high number of variables (high dimensionality) and number of subjects in the database, XGBoost was selected for its abilities to efficiently compute explanations (Doumard et al., 2023). The differential error of the model by age, predicting young individuals being older, or the opposite, was greatly minimized using the custom objective function during XGBoost training (Figure 2b), with no significant impact on the global performance (0.72 and 8.1 on the test dataset for *R* ^2^ and MAE, respectively, Figure 2B).

### 3.2 Estimation of the relative weightsof each variable compared to the whole population or by age group: The global and contextualized explainability of PPA

#### 3.2.1 Model explainability

**FIGURE 3:**
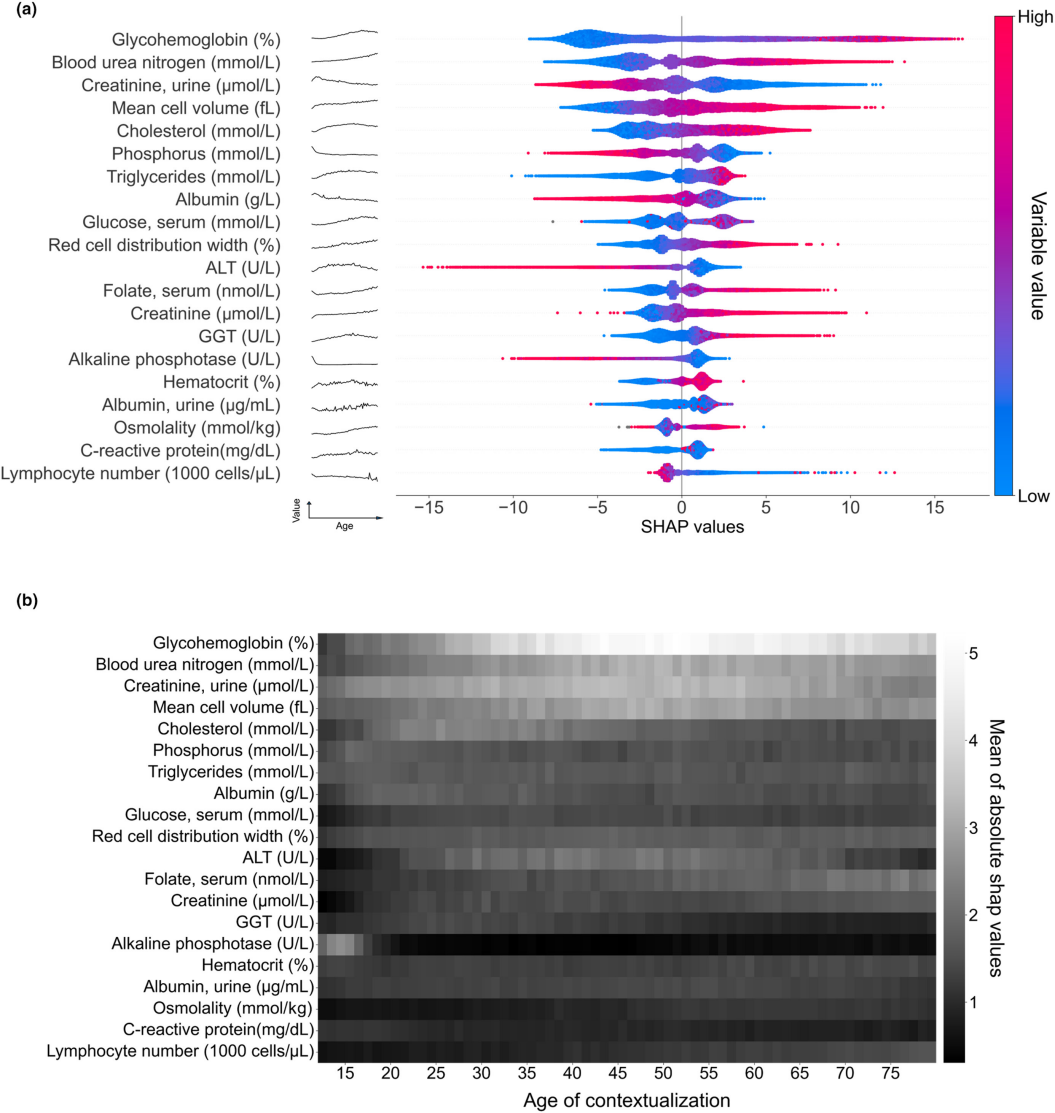
Global and contextualized explainability of physiological age. (a) Global explainability of the PPA model for the top‐20 most important variables (in order of importance based on the mean of absolute SHAP values). Each point color encodes the SHAP value of each variable for each individual; red and blue colors indicate high and low values of the variable, respectively. A positive or negative SHAP value on the x‐axis means that the variable contributed to the positive or negative estimation of physiological age for a given individual. The evolution of the raw variable values over time is depicted as a black line to the right of each variable name. This shows that the evolution of the SHAP values over time does not always follow the evolution of its raw value. (b) Contextualized explainability of the physiological age. SHAP values have been contextualized, taking as a base value the mean predicted age of the individuals with the same chronological age. Heatmap represents the mean of the absolute contextualized SHAP values for each variable (the whiter the color, the higher the mean absolute SHAP value) for each chronological age.

To define the contribution of each variable in individual PPA prediction, the Shapley Additive exPlanations (SHAP) Tree framework was applied on the XGBoost model with Custom Loss model (Doumard et al., 2023). The SHAP value integrates both the effect per se of a given biological variable and the effects of this variable in interaction with other biological parameters. For a given individual (local explanations), the sum of the SHAP values of all variables of the model represents the individual deviation from the mean of chronological age predicted from the entire dataset (39.9 years old in our dataset, i.e., the base value to add to the sum of all SHAP values). The higher the overall SHAP value, the more the variable contributes to the PPA. The summary plot shows the ranking by the mean absolute value of global SHAP contribution for each variable by decreasing importance (Figure S7). The global SHAP values of the top‐20 variables are depicted in Figure 3a, representing 76% of the mean total SHAP sum contribution.

Many of the top‐20 variables were related to metabolism, nitrogen (e.g., uric metabolites and creatinine), carbon (e.g., glycohemoglobin, triglycerides, and glucose), or related to liver function (e.g., albumin, ALT, and GGT). Glycohemoglobin was the biggest contributing parameter (10.7% of the mean total SHAP sum contribution) while serum glucose was ranked 9th (Figure 3a). Urinary and blood creatinine, reflecting renal function, were also shown to contribute to PPA prediction. Several parameters directly or indirectly related to erythropoiesis (mean cell volume, red cell distribution width, hematocrit, and serum folate) were also among the top‐20 variables. Immunity/inflammation (CRP and lymphocyte number) were ranked 19th and 20th, respectively, while other parameters of the immune system (e.g., monocyte or lymphocyte percent, white blood cell count) displayed lower impact on SHAP values (Figure S7, Figure S8). For most of the variables (11 variables over 20), the higher was the variable value, the higher was the deviation from chronological age. No significant difference between males and females in explainability profile and ranking of variables was observed (Figure S9).

#### 3.2.2 Contextualized explainability of PPA (deviations from age‐specific normative data)

While global explainability uses the mean prediction of the whole population as a reference, contextualization refers to the mean prediction of the individuals sharing the same chronological age, in order to overcome putative generational effects. The SHAP contribution of each variable is quoted “contextualized SHAP” in the manuscript. The absolute values of contextualized SHAP values for each variable are presented in Figure 3b. Glycohemoglobin (HbA1c), blood urea nitrogen, mean cell volume, and urinary creatinine proved to contribute throughout life‐course, albeit with a stronger contribution between 40 and 70 years of age. Other variables had more age‐specific contributions, such as alkaline phosphatase (12–18 y.o.), alanine transferase ALT and cholesterol (20–40 y.o.), or lymphocyte number and folate (60 y.o. and over).

### 3.3 Clinical and socio‐economic validation of PPA

#### 3.3.1 Validation on mortality data

We derived the PPA deviation metric, defined for a given individual as the sum of the contextualized SHAP values. Using a multivariate Cox survival model, PPA deviation was found to be a relevant predictor of mortality independently of the chronological age (Table 1). Indeed, a positive PPA deviation value was significantly associated with gradual increase in mortality risk (adjusted hazard ratio with 95% confidence interval, aHR 95%CI 1.18[1.01;1.38], 1.37[1.17;1.59], 1.38[1.18;1.60], and 1.69[1.45;1.97] for the 7th to 10th deciles, compared to the 5th decile, respectively).

**TABLE 1:** Validation on mortality data.

	aHR [95%CI]			
	Complete model	Minimal model	Klemera–Doubal Model	Homeostatic dysregulation
AIC	57,820	57,819	57,607	57,056
Metric deviation (deciles)				
Decile 1	0.80 [0.68;0.94]	0.78 [0.66;0.92]	1.17 [0.91;1.51]	0.57 [0.46;0.72]
Decile 2	0.93 [0.78; 1.09]	0.84 [0.71;0.99]	1.06 [0.83;1.36]	0.62 [0.51;0.76]
Decile 3	0.84 [0.71; 0.99]	0.90 [0.77;1.06]	1.2 [0.97;1.49]	0.90 [0.75;1.07]
Decile 4	0.95 [0.81; 1.11]	0.88 [0.75;1.03]	1.21 [0.99;1.47]	1.09 [0.92;1.29]
Decile 5	1	1	1	1
Decile 6	1.19 [1.03;1.39]	1.21 [1.04;1.40]	1.16 [0.97;1.38]	1.12 [0.95;1.33]
Decile 7	1.20 [1.03;1.39]	1.20 [1.03;1.39]	1.30 [1.1;1.55]	1.45 [1.23;1.69]
Decile 8	1.41 [1.21;1 0.64]	1.25 [1.07;1.45]	1.46 [1.24;1.73]	1.89 [1.62;2.20]
Decile 9	1.29 [1.11;1.50]	1.39 [1.19;1.61]	1.71 [1.45;2.03]	2.7 [2.35;3.15]
Decile 10	1.76 [1.51;2.05]	1.65 [1.41;1.93]	3.26 [2.77;3.83]	3.49 [3.02;4.04]
Gender: Male	0.65 [0.61;0.69]	0.65 [0.61;0.69]	0.59 [0.55;0.63]	0.63 [0.59;0.68]
Age	31.45 [6.23;159]	27 [5.4;138]	74 [15;360]	86 [16;435]
Year of inclusion	1.08 [1.03;1.14]	1.08 [1.02;1.13]	1.11 [0.99;1.07]	1.12 [1.06;1.18]
Age: Year of inclusion	0.998 [0.998;1]	0.998 [0.998;1]	0.998 [0.998;1]	0.999 [0.998;1]

*Note*: Adjusted hazard ratios aHR based on gender, chronological age, and NHANES year of inclusion with 95% confidence interval were computed according to PPA deviation value (the sum of contextualized SHAP values for the complete or minimal model), Klemera–Doubal Model or Homeostatic Dysregulation, taken as deciles. Akaike criterion (AIC) was computed for each model. Subjects at least 18 years old were included in this analysis. An aHR inferior to 1 indicates decreased risk of mortality, and conversely. The lower the AIC, the more the model minimizes the loss of information (the better the model).

#### 3.3.2 Validation on socio‐demographic and medical variables (Table 2)

**TABLE 2:** : Validation on socio‐demographic and medical data.

Variable	*N*	Complete model			Minimal model			Klemera–Doubal Model			Homeostatic dysregulation		
		Coeff.	*p*‐val	dAIC	Coeff.	*p*‐val	dAIC	Coeff.	*p*‐val	dAIC	Coeff.	*p*‐val	dAIC
Socio‐demographic variables													
Gender	***47,168***												
Female	24,294 (52%)	–	–	0	–	–	1243	–	–	11,737	–	–	32,524
Male	22,874 (48%)	1.59	<0.001		1.53	<0.001		1.58	<0.001		−0.09	0.5	
Ethnicity	***47,168***												
Non‐Hispanic white	19,700 (42%)	–	–	0	–	–	1239	–	–	11,686	–	–	32,281
Mexican American	13,017 (28%)	−0.24	0.03		0.02	0.89		−0.29	0.02		1.12	<0.001	
Non‐Hispanic black	9977 (21%)	0.22	0.06		0.06	0.60		−0.94	<0.001		2.89	<0.001	
Others	4474 (9%)	0.93	<0.001		1.12	<0.001		0.92	<0.001		1.10	<0.001	
Family income	***43,137***												
Q1 – Low	11,467 (27%)	–	–	0	–	–	1097	–	–	10,587	–	–	29,832
Q2	13,446 (31%)	−0.12	0.33		−0.06	0.65		0.34	0.02		−0.75	<0.001	
Q3	8294 (19%)	−0.36	0.01		−0.29	0.05		0.35	0.03		−1.24	<0.001	
Q4 – High	9930 (23%)	−0.44	0.001		−0.38	0.006		0.44	0.004		−2.50	<0.001	
Poverty Index Ratio	***43,137***												
No poverty	33,837 (78%)	–	–	0	–	–	1095	–	–	10,596	–	–	29,879
Poverty	9300 (22%)	0.48	<0.001		0.463	<0.001		0.27	0.04		1.88	<0.001	
Medical variables													
Body Mass Index	***46,498***												
Obesity	17,153 (37%)	2.49	<0.001	0	2.50	<0.001	1226	5.20	<0.001	9637	2.97	<0.001	28,645
Overweight	15,397 (33%)	0.94	<0.001		0.90	<0.001		1.87	<0.001		0.20	0.20	
Normal weight	13,175 (28%)	–	–		–	–		–	–		–	–	
Underweight	773 (2%)	−0.31	0.38		−0.50	0.17		−2.12	<0.001		0.80	0.10	
Tobacco exposure	***47,046***												
No exposure	34,904 (74%)	–	–	0	–	–	1225	–	–	11,662	–	–	32,487
Exposure	12,142 (26%)	0.74	<0.001		0.86	<0.001		−0.94	<0.001		0.93	<0.001	
Sedentarity	***17,207***												
Active	15,380 (89%)	–	–	0	–	–	300	–	–	2451	–	–	4305
Sedentary	1827 (11%)	0.92	<0.001		0.81	0.001		1.29	<0.001		2.03	<0.001	
Pathologies													
Liver diseases	1750 (4%)	0.90	<0.001	0	0.74	0.003	1290	4.06	<0.001	11,231	2.70	<0.001	31,926
Coronary Heart Diseases	4186 (9%)	2.27	<0.001	0	2.31	<0.001	1274	4.10	<0.001	11,123	4.30	<0.001	31,217
Diabetes	8079 (17%)	5.85	<0.001	0	6.04	<0.001	1161	8.80	<0.001	9936	8.19	<0.001	32,561
Thyroid diseases	4049 (9%)	1.25	<0.001	0	1.23	<0.001	1283	2.25	<0.001	11,328	0.86	<0.001	31,421
Arthritis	11,681 (26%)	1.66	<0.001	0	1.71	<0.001	1271	2.43	<0.001	11,281	1.77	<0.001	31,460
Cancer	3450 (8%)	0.84	<0.001	0	0.82	<0.001	1275	0.73	<0.001	11,332	0.39	0.13	30,907
Kidney diseases	1304 (3%)	4.84	<0.001	0	4.86	<0.001	1284	13.3	<0.001	10,041	8.99	<0.001	31,213
Bronchitis	7877 (17%)	0.83	<0.001	0	0.69	<0.001	1258	1.60	<0.001	11,645	1.42	<0.001	32,500
Auto‐immune digestive disease	59 (1%)	1.69	0.19	4376	1.69	0.20	4551	0.55	0.68	4439	−0.84	0.30	0
Digestive ulcer	388 (10%)	0.34	0.50	2819	0.16	0.76	2895	0.47	0.42	3835	0.14	0.70	0
Eye disease	451 (8%)	0.86	0.09	3956	0.70	0.18	4188	2.29	<0.001	5263	1.15	0.002	0
Dermatologic disease	842 (4%)	0.04	0.91	15,899	−0.06	0.88	16,313	−0.08	0.83	19,516	−0.002	0.99	0
AAC24 score	***2770***												
Low score	2136 (77%)	–	–	1525	–	–	1606	–	–	2226	–	–	0
Med score	219 (8%)	2.20	0.003		1.99	0.009		2.14	0.01		2.93	<0.001	
High score	415 (15%)	1.58	0.004		1.54	0.006		0.79	0.20		0.09	0.83	

*Note*: For each model (complete model, minimal model, Klemera–Doubal Model (KDM), or Homeostatic Dysregulation (HD)), a multivariate model adjusted on chronological age and gender was performed and the coefficient and its associated *p*‐value regarding the validation data were presented. Akaike criterion (AIC) was computed for each model and presented as the difference compared with the best model (dAIC). Subjects at least 18 years old were included in this analysis.

The regression coefficient is the PPA deviation contribution to validation variable, adjusted for chronological age and gender. Altered health condition or being in a socially disadvantaged population were significantly associated with increased PPA deviation for a majority of variables. Being a male, poor, exposed to tobacco, obese, sedentary or with a systemic disease was associated with a significantly increased PPA deviation values. Of note, having high family income was significantly associated with a lower PPA deviation value.

#### 3.3.3 Comparison to KDM and homeostatic dysregulation (HD) metrics (Table 2)

To further validate PPA, KDM and HD metrics were computed using the same set of variables. As revealed by a lower Akaike information criterion (AIC), PPA better fits with socio‐demographic variables and most of medical variables than KDM and HD. For mortality, HD metric achieved the lowest AIC. However, PPA also successfully captured mortality, with a decreased mortality risk for a negative PPA value, and conversely.

### 3.4 Impact of variations of each variable on the SHAP explanations: A range of biological values associated with healthy aging

**FIGURE 4:**
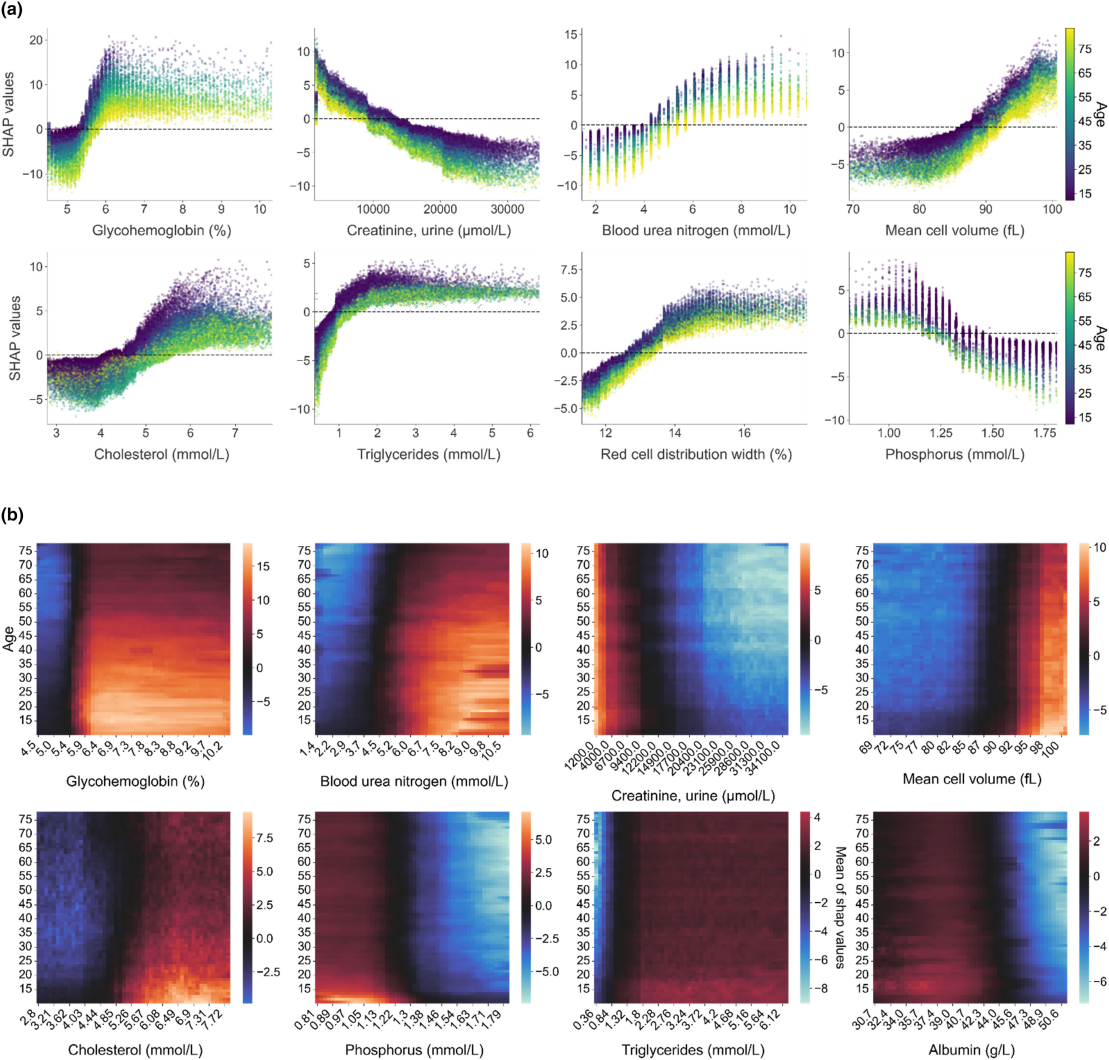
Partial Dependence Plots of contextualized SHAP values. (a) Contextualized SHAP values as a function of variable values for the top‐8 variables. Each dot represents an individual. The color indicates the corresponding chronological age (scale on the right). X‐axis corresponds to the real value of the variable, while the y‐axis corresponds to the SHAP value given to this individual for this variable. The dotted line corresponds to a SHAP value of 0, which means that when the individual displays a variable value for which the SHAP value is 0, the variable has no impact on the physiological age. (b) Heatmap of contextualized SHAP values as a function of chronological age. The color of each pixel indicates the average SHAP value of a variable (x‐axis) as a function of chronological age (y‐axis). An example of interpretation is illustrated in Figure S3.

Partial dependence plots revealed the impact of one variable on the PPA by averaging the influence of all other variables (Figure 4a; Figure S10a). Curve shapes that were similar between ages clearly revealed the different ranges of the variable value for which the corresponding contextualized SHAP values were positive, neutral, or negative. For example, while the contextualized SHAP values were negative for low glycohemoglobin, a sharp increase occurred for values in the 5%–6% window, confirming the accuracy of the follow‐up value (Figure 4a; Figure S3a). This transition zone, characterized by crossing the zero‐line between age scales, was different according to age groups. This was visualized as a dark zone in the heatmap in Figure 4b and Figure S10b. Thus, while the threshold of 5.4% characterized a border for young subjects, it evolved with age, increasing to 5.8% for subjects older than 50 (Figure 4b; Figure S3b). This produces biological alert thresholds, adaptable to chronological age of a subject. Similar analysis can be applied to all variables (Figure 4a). Figure 4b underscores the decrease in the normal range of biological values with age. Altogether, these results show the contribution and relevance of contextualized SHAP values to define a new metric and standard suitable to define a physiological health status for all age groups.

### 3.5 Identification of different aging trajectories by clustering profiles of identical contextualized explanations

**FIGURE 5:**
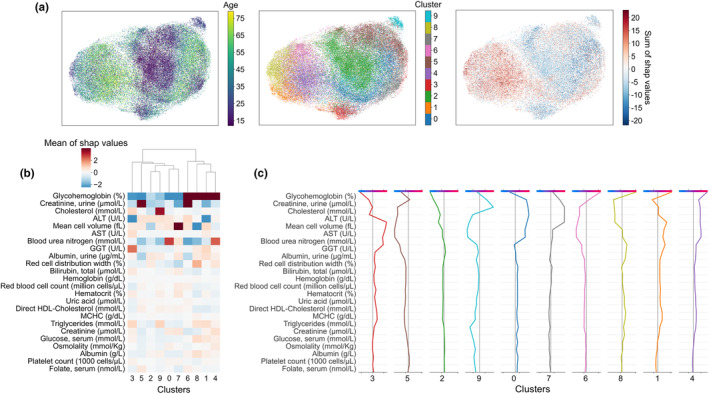
Clustering SHAP values to reveal healthy aging trajectories. Individuals were clustered by agglomerative clustering based on their contextualized SHAP values. Chronological age was not added as a clustering variable (a) UMAP 2D‐projections were colored by chronological age, identified cluster and sum of contextualized SHAP values from left to right, respectively. It is thus possible to see, according to the age distribution of the different clusters, profiles of individuals with accelerated aging. (b) Signature of each cluster for the 24 variables allowing significant distinction between at least 2 clusters. The heatmap shows the average value of each variable for each cluster. (c) Decision plot profile for each cluster. Starting from the bottom, the cumulative contribution of each variable was presented (in positive and negative values) to the predicted final value (at the top of the diagram). For each cluster, we indeed have the “average” individual representative of the cluster.

To identify putative pathways linked to aging, all contextualized SHAP values were clustered, irrespective of chronological age (Figure 5a). Ten SHAP clusters grouped into two classes according to the glycohemoglobin SHAP value were identified. This suggested that profiles corresponding to the same PPA deviation involved different physiological pathways supporting aging. Clustering was strongly driven by the SHAP values of glycohemoglobin. Contribution of low (below clinical threshold at 6%) glycohemoglobin appeared to correlate with a “younger physiology” in older individuals. Within classes corresponding to positive and negative SHAP values of glycohemoglobin, changes in a limited set of variables (urinary creatinine, cholesterol, ALT, mean cell volume (MCV), aspartate transferase (AST), blood urea nitrogen (BUN), gamma‐glutamyl transferase (GGT)) distinguished the clusters. All other variables weakly contributed to the PPA estimation (Figure 5b,c). Clusters 2 and 4 were characterized by a systematic negative and positive PPA deviation of key biological variables (Figure 5c). All other profiles were characterized by a mix of positive and negative SHAP values of the same key variables.

### 3.6 Generation of a minimal model by recursive feature elimination

**FIGURE 6:**
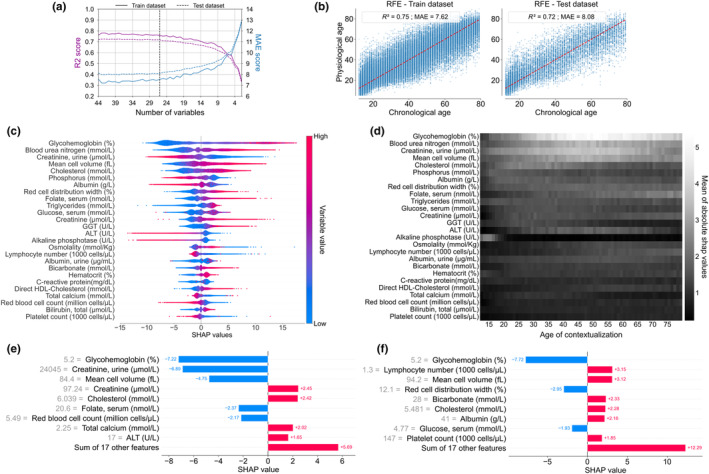
Generation of a minimal model to estimate physiological age (RFE model). (a) Evolution of XGBoost custom loss model performance (*R* ^2^ and MAE scores) through recursive feature elimination (RFE). A model with 26 variables seems sufficient without significantly altering the model performance. Global explainability of the PPA model. (b) Graphical representation of the predicted physiological age defined by the RFE model (based on XGBoost with Custom loss) as a function of chronological age, on the train and test datasets. The red line indicates situations where physiological age is identical to chronological age. (c) Global explanation of the RFE model with the mean of absolute SHAP values in order of importance. Each point color encodes the SHAP value of each variable for each individual, red and blue colors for high and low values of the variable, respectively. On the x‐axis, a positive or negative SHAP value means that the variable for one individual contributes to a positive or negative estimation of physiological age relative to chronological age. (d) Heatmap of the mean of the absolute contextualized SHAP values for each variable (the whiter the color, the higher the mean absolute SHAP value) for each chronological age. (e, f) For a given individual, a personalized and contextualized explanation of physiological age wan be given. To the base value (mean predicted age of the individuals of the same chronological age), several contributions of each variable contextualized SHAP value were added to obtain the physiological age (increase of PPA in red, decrease of PPA in blue). (e) Example of an individual of 61 y.o predicted 49, (f) and an individual of 64 y.o. predicted 76.

In order to test the robustness of the model with a perspective of PPA application to the general population, we iteratively eliminated variables one by one. The run‐out RFE algorithm (Figure 6) indicated that 26 variables were sufficient to predict PPA without significantly decreasing the performance of the model estimated by the *R* ^2^. Similar to the complete model, this minimal model was also predictive of mortality, and fitted well with socio‐demographic and medical variables (Tables 1; 2).

## 4 DISCUSSION

Personalized estimation of physiological aging requires the capture of subtle physiological changes/dysfunctions as early as possible before any clinical manifestation. Such an objective is highly challenging (Jylhävä et al., 2017; Ferrucci et al., 2020) and deviates from the traditional medical approach where interventions mostly occur in the presence of clinical manifestations.

Using an innovative explainable ML pipeline with non‐supervised selection of biological variables, we defined personalized physiological age (PPA) as a predictor of mortality and lifespan, as well as a risk factor for chronic diseases. PPA framework also identifies the relative and precise quantitative weight of variables contributing to its estimation from the earliest age. PPA gives accelerated or delayed aging relative to specific aging profiles. Because PPA is derived from biological variables routinely available, it represents an efficient and cost‐effective tool for general populations. Furthermore, the PPA framework can be easily translated to address other clinical issues and to quantify the relevance of new biomarkers with an accurate associated metric.

For complex systems such as physiological aging, the use of ML appears particularly suitable to capture, whatever their nature and intensity, complex interactions among a wide type of variables. Although some studies have used ML to define physiological age (Sun et al., 2021), these models are black‐boxes lacking explainability, hence preventing the ability to check for reliability and model‐inferences (Diprose et al., 2020; Linardatos et al., 2021). An exception is a very recent report on the prediction of individual trajectories and survival combining machine learning and interaction network (Farrell et al., 2022). Future developments could also consider tree‐based glass‐box models such as Explainable Boosting Machine (Lou et al., 2013) or feature attribution methods for neural networks (Janizek et al., 2021; Lombardi et al., 2021). Explainability better sustains evidence‐based acceptability and could allow for data‐driven personalized medical‐management (Stiglic et al., 2020). To achieve this goal, we developed an explainable PPA estimation where (1) all subgroups were represented without significant imbalance, (2) adequate ML techniques were able to consider performance discrepancy across the age group (custom loss), (3) the training data (source population) were representative of the target population by creating a test dataset and minimizing overfitting.

For PPA explainability, we used state‐of‐the‐art explainable techniques, such as the SHAP algorithm, to compute local explanations at the individual level. This integrates the effects of the biological variable per se, but also the effects of interactions between variables (Doumard et al., 2023). Compared to other indexes, PPA performs better to capture socio‐demographic disparities and medical conditions than KDM or homeostatic dysregulation (HD), and better capture mortality than KDM. The global and contextualized SHAP analyses are similar to HD and KDM approaches, respectively, but in addition allow capture of complex interactions (including non‐linear ones) between variables, contrary to classical biostatistical approaches. Contextualized SHAP strategies reduce the risk of possible generational bias since in other studies, age predictors suitable for one group may not operate for other groups (Sagers et al., 2020). The difference between global and contextualized analysis can be illustrated using alkaline phosphatase (ALP). In a global approach, ALP makes sense over age, while in a contextualized approach it is meaningful at a young age, certainly in accordance with a role in bone development (Sekaran et al., 2021). All SHAP value analyses highlight the major contribution of HbA1c to PPA explainability, as well as the identification of aging clusters. This is consistent with its biogenesis reflecting disturbance in glucose homeostasis affecting multiple pathways over long intervals (Little et al., 2019; Ravera et al., 2019; RaviKumar et al., 2011). More indirectly, it connects to energy homeostasis control and carbohydrate metabolism in organisms (Wilson & Matschinsky, 2021). Recently, a genome wide analysis for polygenic traits has highlighted the high heritability of HbA1c value, which strengthen the value of this parameter in reflecting glucose metabolic regulation, and thus its importance in contributing to the determination of biological age (Weiner et al., 2023). Being more strongly involved in PPA than glycemia itself, HbA1c is a paradigm suggesting that indirect and cumulative parameters might be more relevant to assess physiological age than regulated variables, changes in which reveal pathology at a late stage of dysfunction. Given the importance of HbA1c, it may be appropriate to use this variable in complement to the first 10–15 variables that contribute significantly to PPA not only for monitoring diseases but also as a signal for subclinical events as shown by contextualized SHAP. The general involvement of metabolism is also emphasized in the SHAP clusters by the differentiating weight of variables related to red blood cells, liver (ALT, AST, and GGT), and kidney (BUN and urinary creatinine). The centrality of metabolism was previously reported (Hao et al., 2021), together with that of the liver/kidney axis, both in line with previous findings (Ahadi et al., 2020) proposing to stratify individuals into different ageotypes using four groups of pathways: liver and kidney dysfunctions, immunity pathways, metabolism, and inflammation. The number and the weight of variables related to red blood cells is reminiscent of the importance of such multistep, finely‐tuned process gradually affected in aging. Except for lipid metabolism, no variable seems to be clearly related to cardiovascular system. Because heart and kidney variables are strongly correlated (Hao et al., 2021), the PPA algorithm could have selected a single class of variables bringing similar relevant information. Alternatively, chronic inflammation being major contributor of cardiovascular alteration, ultrasensitive‐CRP could be more informative. As for HbA1c, the selection of urinary metabolites by the algorithm could also be explained by the consideration that their evolution reflects a cumulative temporal effect. Surprisingly, immune components have a low influence on PPA and appear in the small but multiple adjustments associated with immune variables. These variables are included in top‐20 of variables for global explainability, but not in contextualized ones that described the relative evolution compared to contemporaries. This could be explained by the very limited number of immunologic variables and/or the fact that the immune variable available in routine clinical testing is not very informative for estimating physiological aging because the selected variables classically measured allow the identification of immune responses to acute aggressions rather than small deviations.

Although a limited number of variables carry weight in the different profiles, the low range involvement of many other biological variables in the PPA estimation perfectly illustrates the complexity of the physiological networks and the importance to detect subclinical signals. This also highlights the need for a combination of biomarkers for an accurate physiological age estimation (Cohen et al., 2015). Even when findings are consistent with the literature (i.e., the role of energy metabolism), the PPA explainable model allows further insight, allowing for a precise analysis of the positive or negative contribution to PPA of each metabolism‐related variable with a high level of refinement. The partial dependence plots not only reveal the evolution of the contribution to the PPA of each variable with age, but also provide precise information about the contribution of a given variable to PPA according to its raw value. Beyond the shape of the profiles, which indicates proportionality (linear contribution) or a shift (sigmoid‐like contribution) in the importance of the variables, the remarkable result is the unsupervised identification of critical ranges of precise values for each variable, whether it is widely characterized (such as HbA1c) or not. Whatever the profiles of contextualized partial dependence plots, the contextualized SHAP values are consistent with a natural age‐related drift of biological parameters. This age‐dependent adjustment of referent value for each variable should lead to the early and accurate identification of small changes anticipating future dysfunction tissue/organ dysfunction and intrinsic capacity of decline.

The clustering of the contextualized SHAP values reveals different individual physiological age deviation profiles with specific variable patterns, overcoming the natural drift in balance of biological parameters with age. In other words, PPA would support identification and deciphering of pathophysiological profiles at risk of accelerated aging. In the context of medical care, this makes it possible to identify sub‐categories of patients and their underlying hierarchy that could be used to implement targeted follow‐up and care. In a research and development context, the analysis of these profiles, with additional biological parameters, will make identify new pathophysiological targets for early identification and management of age‐related dysfunctions.

Some limitations may be mentioned in the present study. First, the NHANES study is based on a USA‐based population cohort and reflects the impact of a certain type of regimen and socio‐economic environment on aging. This hampers generalization, making it necessary to validate the metric on other populations from other countries, although physiological dysregulations appear stable across European and North American populations (Cohen et al., 2014) and even primates (Dansereau et al., 2019). The use of this large database suggests that the model could be used on any consistent database. Cross‐sectional in nature, the use of NHANES data prevents from inferring causalities of results, even though several studies are reasonably supporting. Similar investigations performed on longitudinal studies will bring additional data that better defines the pathophysiological consequences on healthy aging and particularly on frailty status (Ahadi et al., 2020; Elliott et al., 2021). Of course, the precise causal link and the types of interactions between the variables remain to be definitively established.

The use of a limited number of biological variables from standard biochemical analyses paves the way for routine medical use of PPA with a minimum investment. However, the methodology of this study should not be seen as an end, but as a general and evolving framework, where biomarkers can be implemented to improve and precisely identify age‐related imbalances. Variables related to the architecture and the structure of the tissue could be particularly appropriate to identify a drift in architecture/function relationship as we recently proposed (Kemoun et al., 2022). This work does not take into account the biological/mechanical relationships between the different variables. Of note, the low relative importance of immunity parameters may be due to a lack of relevant variables in the dataset or reflect the fact that inflammatory component, defined as inflammaging, could be masked by another variable. Also, variation of low range CRP values, which are widely used in the follow‐up of cardiological patients, was not accessible in the dataset. It is noteworthy that the serological status was not available in the dataset and hence not considered, while it is now well accepted that most chronic infectious diseases have direct and indirect, treatment‐related, impacts on aging trajectories. In the present study, inflammatory markers could address part of these impacts (Ray & Yung, 2018). In the future, the inclusion of new parameters revealing a cumulative effect such as HbA1c should improve the precision of the estimation and thus the recognition of smaller age‐related deviations.

In a nutshell, this work, using standard biological variables, provides not only a practical and powerful tool ready to use in medical care while remaining scalable with integration of other biomarkers, but also a complete explainable ML framework to quantitatively decipher the basis of complex physiological phenotypes. At the crossroads between biology, epidemiology, and informatics, this work offers both the opportunity to reflect on pathophysiology, a predictive tool for precision medicine, and a study framework that can be extended to many other topics.

## AUTHOR CONTRIBUTIONS

Conceptualization: D.B., I.A., P.K., L.P., P.M., and L.C.; methodology: D.B., E.D., I.A., J.A., C.D., L.P., P.M., and L.C.; software: D.B., E.D., and P.M.; validation: J‐C.P., S.C‐B., J.A., C.D., and L.P.; formal analysis: P.M. and P.K.; investigation: D.B., E.D., and P.M.; data curation: D.B., E.D., F.F., and P.M.; writing—original draft preparation: P.K., P.M., and L.C.; writing—review and editing: D.B., E.D., F.F., I.A., J‐C.P., S.C‐B., J.A., C.D., L.P., P.M., and L.C.; supervision: P.M. and L.C. All authors have read and agreed to the published version of the manuscript.

## CONFLICT OF INTEREST STATEMENT

The authors declare no competing interests.

## Supporting information


Data S1.

## Data Availability

The data that support the findings of this study are available from the corresponding author upon reasonable request.
