# Supplementary material for: Explainable machine learning framework to predict personalized physiological aging
Source: Aging Cell. 2023 Jun 10;22(8):e13872. doi: 10.1111/acel.13872 (PMC10410015; doi:10.1111/acel.13872)
Supplement: Supplementary file 1 — Data S1. [file ACEL-22-e13872-s001.pdf]

Supplementary Data

Explainable Machine Learning Framework to Predict Personalized Physiological Aging

David Bernard, Emmanuel Doumard, Isabelle Ader, Philippe Kemoun, Jean-Christophe Pages, Anne Galinier, Sylvain Cussat-Blanc, Felix Furger, Luigi Ferrucci, Julien Aligon, Cyrille Delpierre, Luc Pénicaud, Paul Monsarrat, Louis Casteilla

List of supplementary data

Supplementary Text....2

Fig. S1 : Screenshots of the dedicated interface that has been used to annotate the database....3

Fig. S2: Distribution of data within the final dataset....4

Fig. S3: Interpretation of partial dependance plot (A) and heatmap of contextualized SHAP values (B). 5

Fig. S4: Gender and chronological age distribution of individuals 5

Fig. S5: No gender and age group imbalance between train and test datasets 6

Fig. S6 : Distribution of residuals for the prediction of chronological age by Random Forest, Decision Tree and Elastic Net. 6

Fig. S7: Relative importance of the most important variables in physiological age prediction. 7

Fig. S8: Global explainability of the PPA model in importance order of mean of absolute SHAP values. 7

Fig. S9: Global explainability of the PPA model in importance order of mean of absolute SHAP values for male (A) and female individuals (B). 8

Fig. S10: Partial Dependence Plots of contextualized SHAP values....9

Table S1: List of the 48 biological variables, by alphabetical order 11

Table S2: List of hyperparameters used during model tuning....12

Table S3: Details of the validation variables....13

Supplementary Text

Variable selection and merging

To generate a consistent and large database, with a maximal number of common biological variables for subjects, we performed a manual data cleaning to eliminate redundant outcomes, both within the same year and in different years. This step requires human intervention. For instance, some variables hold different labels and/or codes across years (*e.g.*, LBDHDLSI, “HDL-cholesterol (mmol/L)” from 1999 to 2002 and LBDHDDSI, “Direct HDL-Cholesterol (mmol/L)” from 2003 to 2018), or have different units (*e.g.*, serum glucose in mmol/L LBDSGLSI and in mg/dL LBXSGL). To optimize cleaning by investigators (IA, LC, LP, PK and PM) and ensure reproducibility, a web interface was developed (Fig. S1).

For each laboratory variable available, investigators were independently asked to select the variables based on the inclusion criteria described above. A similarity algorithm (using cosine similarity and Levenshtein distance) based on the “SAS” and “text” labels, proposed a list of potentially synonymous terms to investigators. A manual search tool with autocompletion was also available.

When identical variables were measured several times, the mean value was considered (for example*:* both variables LB2NEPCT and LBXNEPCT corresponded to the same biological variable, *i.e.* segmented neutrophils percent). Biological variables expressed in international units (SI) were privileged over their non-SI counterparts. In case of disagreement by an investigator, a collegial decision was made at the consensus phase. After this step, and considering the distribution of the number of available variables for a given number of subjects, the largest dataset with the minimum amount of missing data was defined. The cut-off for this distribution selected variables with at least 50,000 individuals. Individuals with more than 10% missing values were also dropped from database. After processing, the selected dataset contained 60,322 individuals with 48 laboratory variables (Table S1) and limited missing data (0.6% of data, Fig. S2).

A

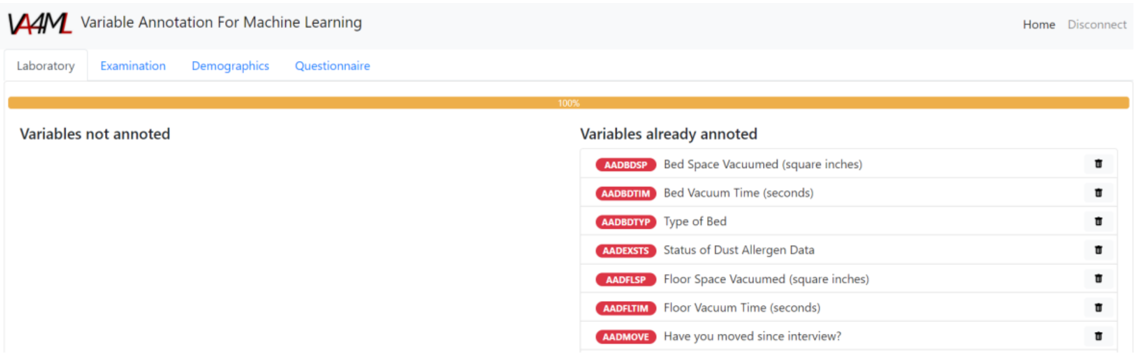

B

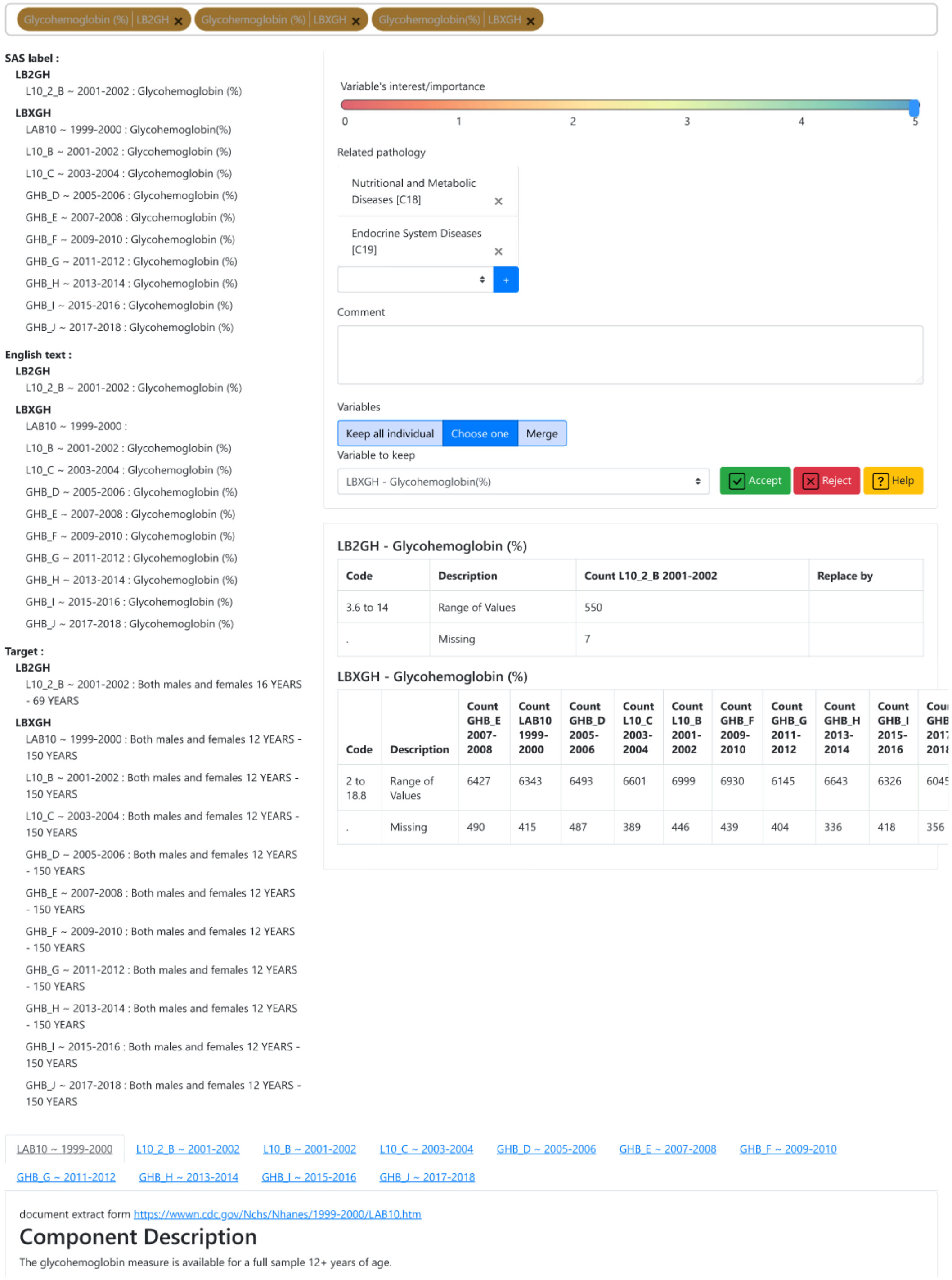

Fig. S1 : Screenshots of the dedicated interface that has been used to annotate the database.

Each user had his own account and the laboratory variables **(A)** were selected, grouped and/or annotated **(B)**.

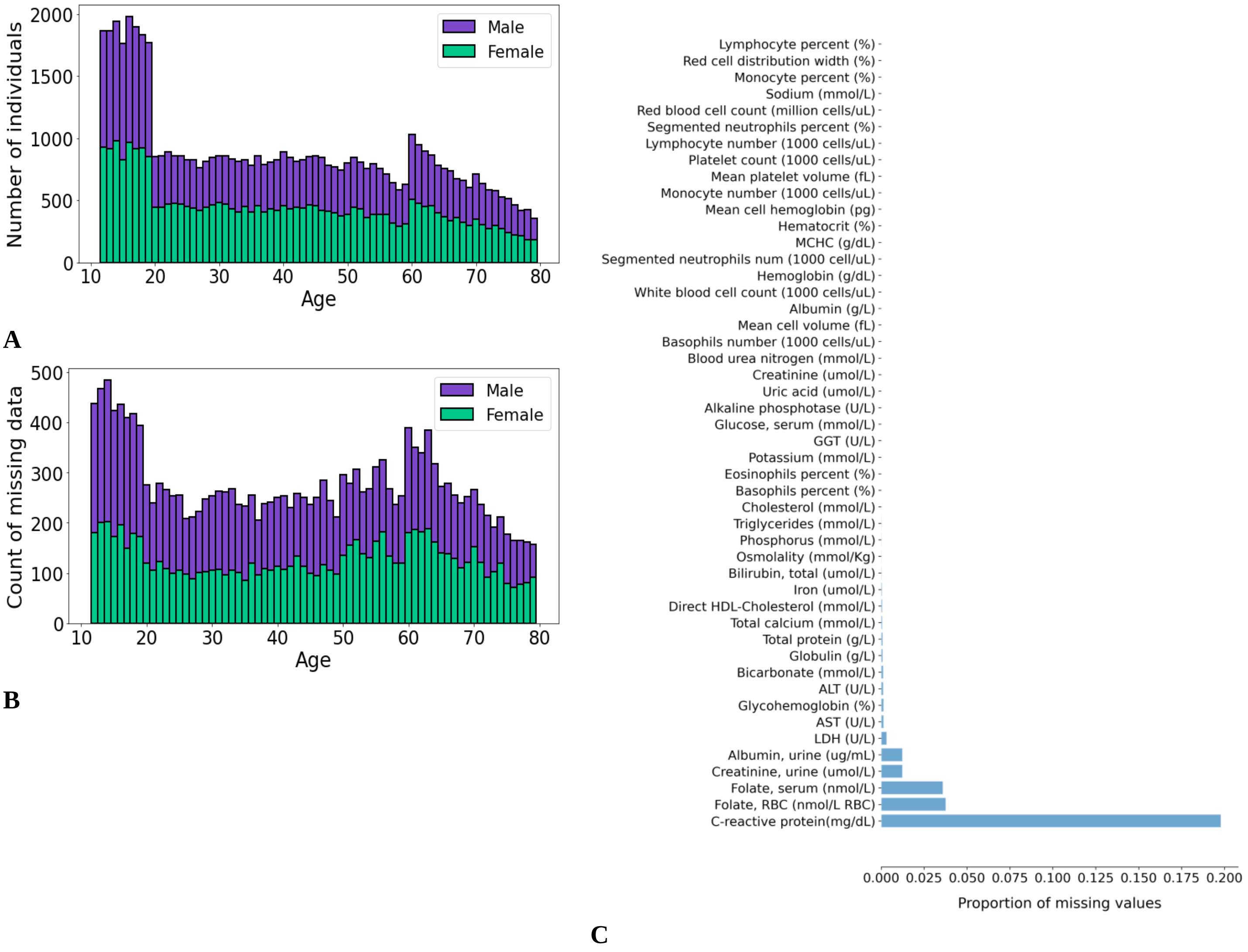

Fig. S2: Distribution of data within the final dataset.

(A) Distribution of the number of individuals by chronological age and gender. The amount of data from 12 to 20 years was twice those of other age and a 25% decrease of available subject number from 70 to 79 years old. No major gender imbalance was pointed out across age groups. **(B)** Uniform distribution of missing data among chronological age and gender. **(C)** Proportion of missing data by variable. The amount of missing data was low (25% of individuals with one missing value representing 0.6% of the total values). They were mainly related to the lack of C-reactive protein, folate, albumin, and creatinine data.

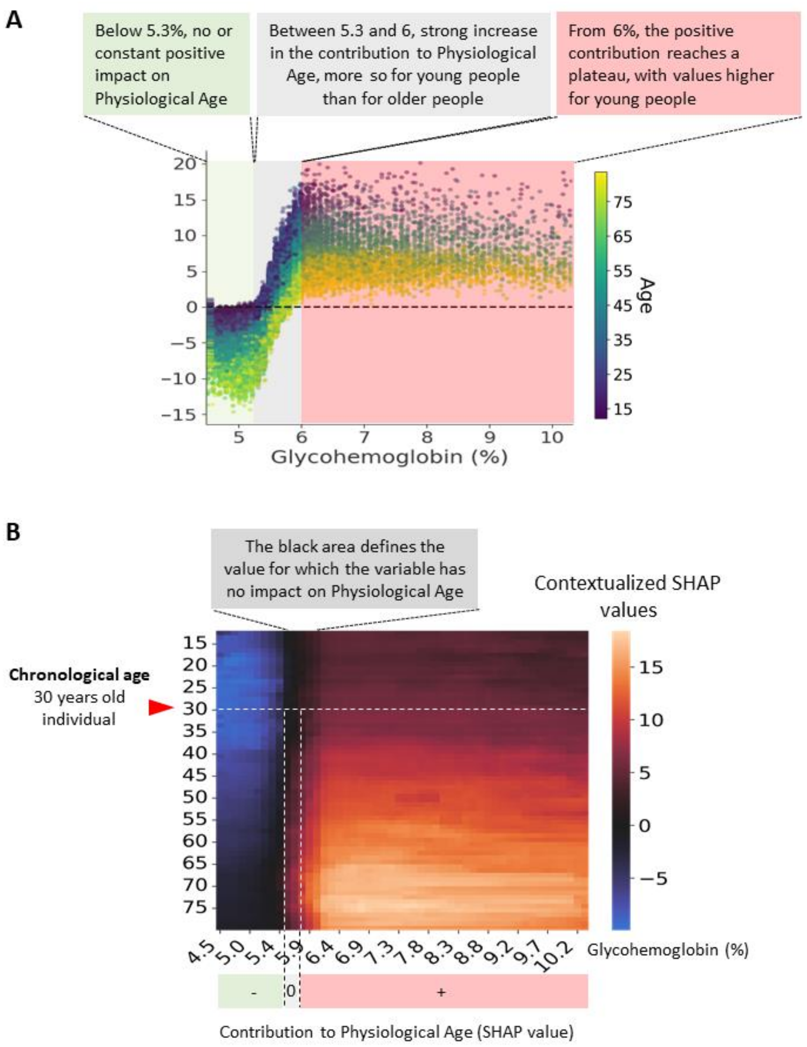

Fig. S3: Interpretation of partial dependance plot (A) and heatmap of contextualized SHAP values (B).

(A) Each dot represents an individual. The color indicates the corresponding chronological age. X-axis corresponds to the real value of the variable, while the y-axis corresponds to the SHAP value given to this individual for this variable. While the contextualized SHAP values are negative in low values of glycohemoglobin, a sharp increase occurs between 5.3 and 6%. This transition zone, characterized by the passage from zero, is different according to age and clearly visualized in (B) as a dark zone in the heatmap. **(B)** Heatmap of contextualized SHAP values as a function of chronological age. The color of each pixel indicates the average SHAP value of a variable (x-axis) as a function of chronological age (y-axis). For an individual of 30 y.o., the normal range is about 5.5 – 5.8%.

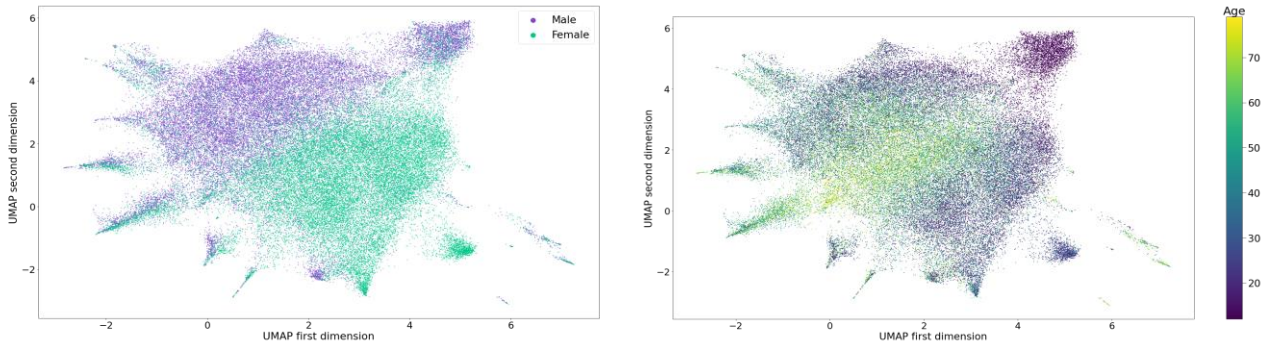

A **B**

Fig. S4: Gender and chronological age distribution of individuals

UMAP 2-dimensional projection of the 48 variables of the dataset, colored by chronological age **(A)** and gender **(B)**. UMAP revealed some clustering across the second dimension by gender with mostly males in the upper part of the UMAP and females in the lower part. In addition, the first dimension mainly contains chronological age information, with a gradient from youngest to oldest from right to left.

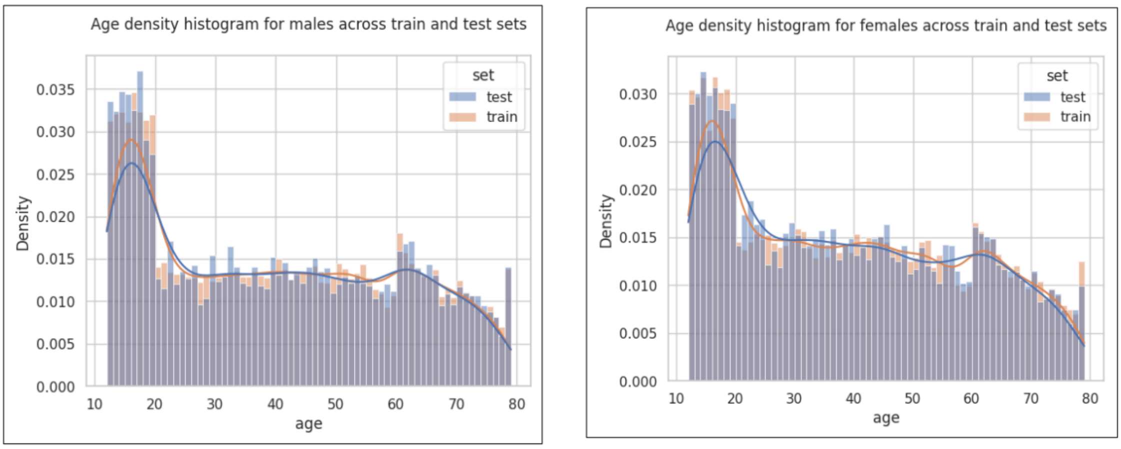

Fig. S5: No gender and age group imbalance between train and test datasets

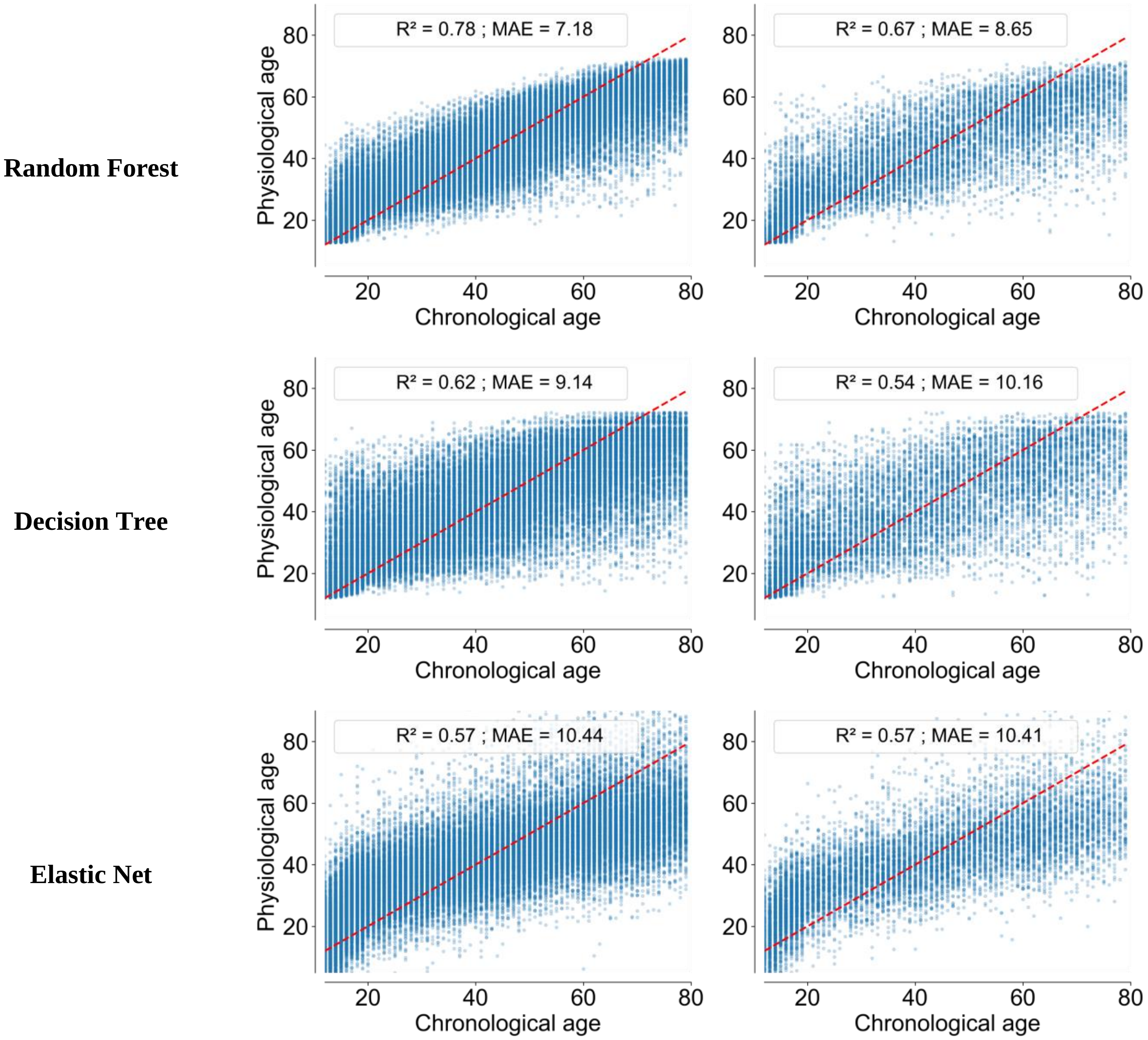

Fig. S6 : Distribution of residuals for the prediction of chronological age by Random Forest, Decision Tree and Elastic Net.

Left scatter plot illustrates the distribution of residuals on the train dataset and right scatter plot on the test dataset. The performances are largely inferior to those obtained with XGBoost or MultiLayer Perceptron with a strong performance discrepancy across the age group (younger people are predicted to be older and conversely).

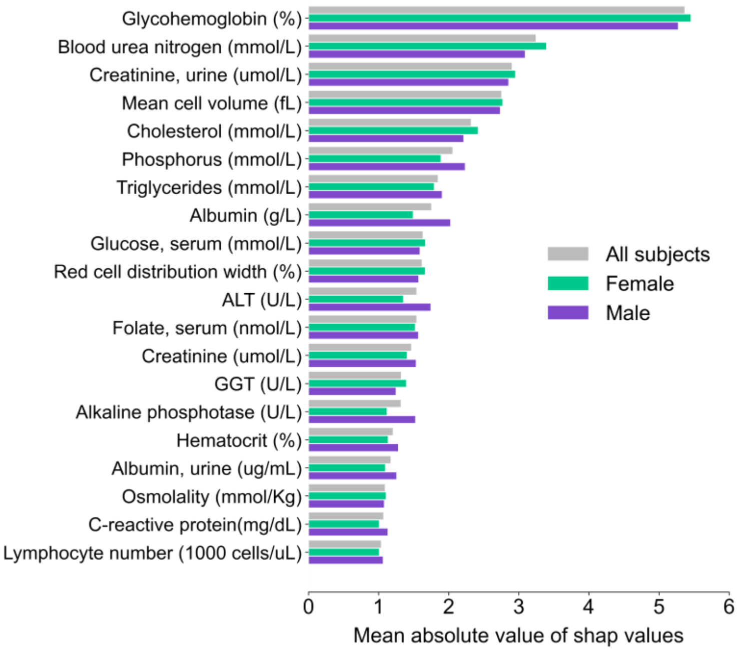

Fig. S7: Relative importance of the most important variables in physiological age prediction.

The mean absolute value of shap values for the 20 most important variables are shown for the whole population (gray), female (green) and male (purple) populations. A similar importance can been shown according gender.

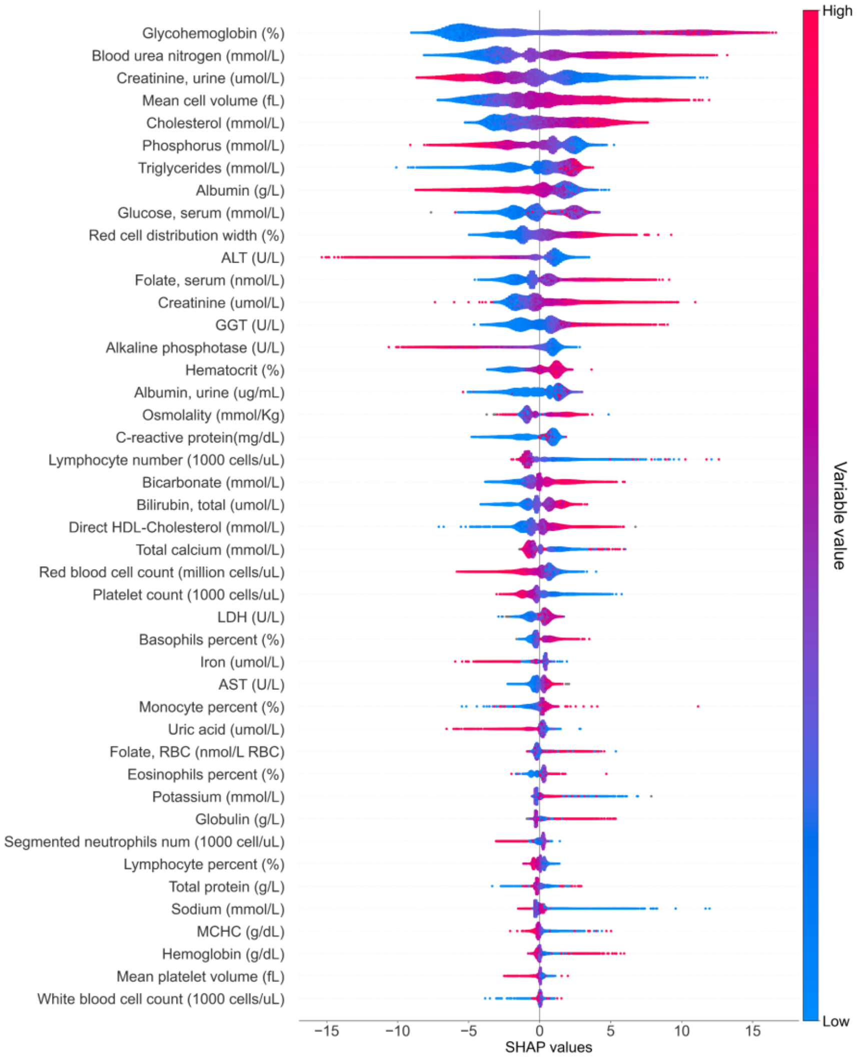

Fig. S8: Global explainability of the PPA model in importance order of mean of absolute SHAP values.

Each point color encodes the SHAP value of each variable for each individual, red and blue colors for high and low values of the variable respectively. On the x-axis, a positive or negative SHAP value means that the variable, for one individual contributes to the estimation of physiological age positively or negatively respectively.

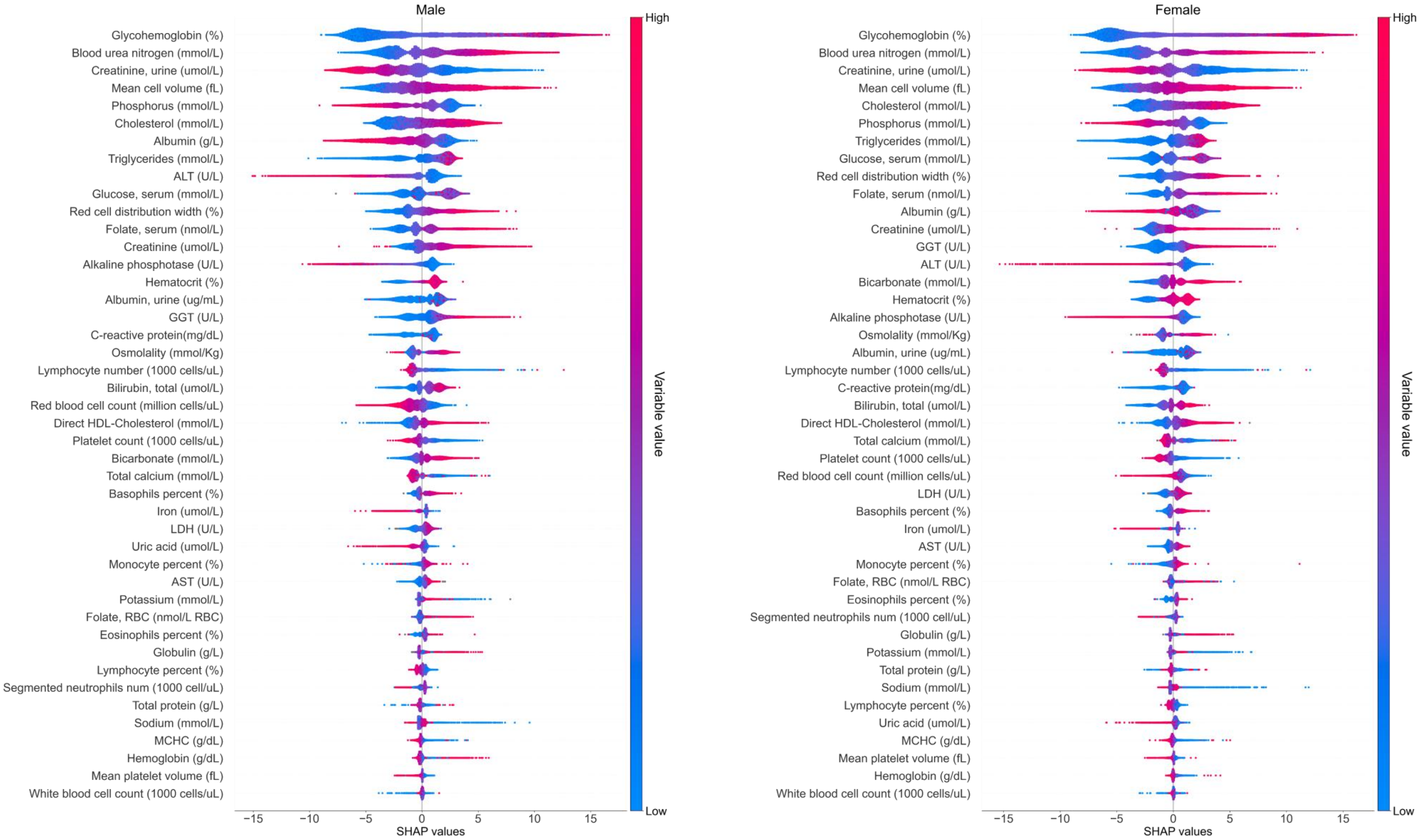

Fig. S9: Global explainability of the PPA model in importance order of mean of absolute SHAP values for male (A) and female individuals (B).

Each point color encodes the SHAP value of each variable for each individual, red and blue colors for high and low values of the variable respectively. On the x-axis, a positive or negative SHAP value means that the variable, for one individual contributes to the estimation of physiological age positively or negatively respectively. Similar explainability profile can be found between male and female.

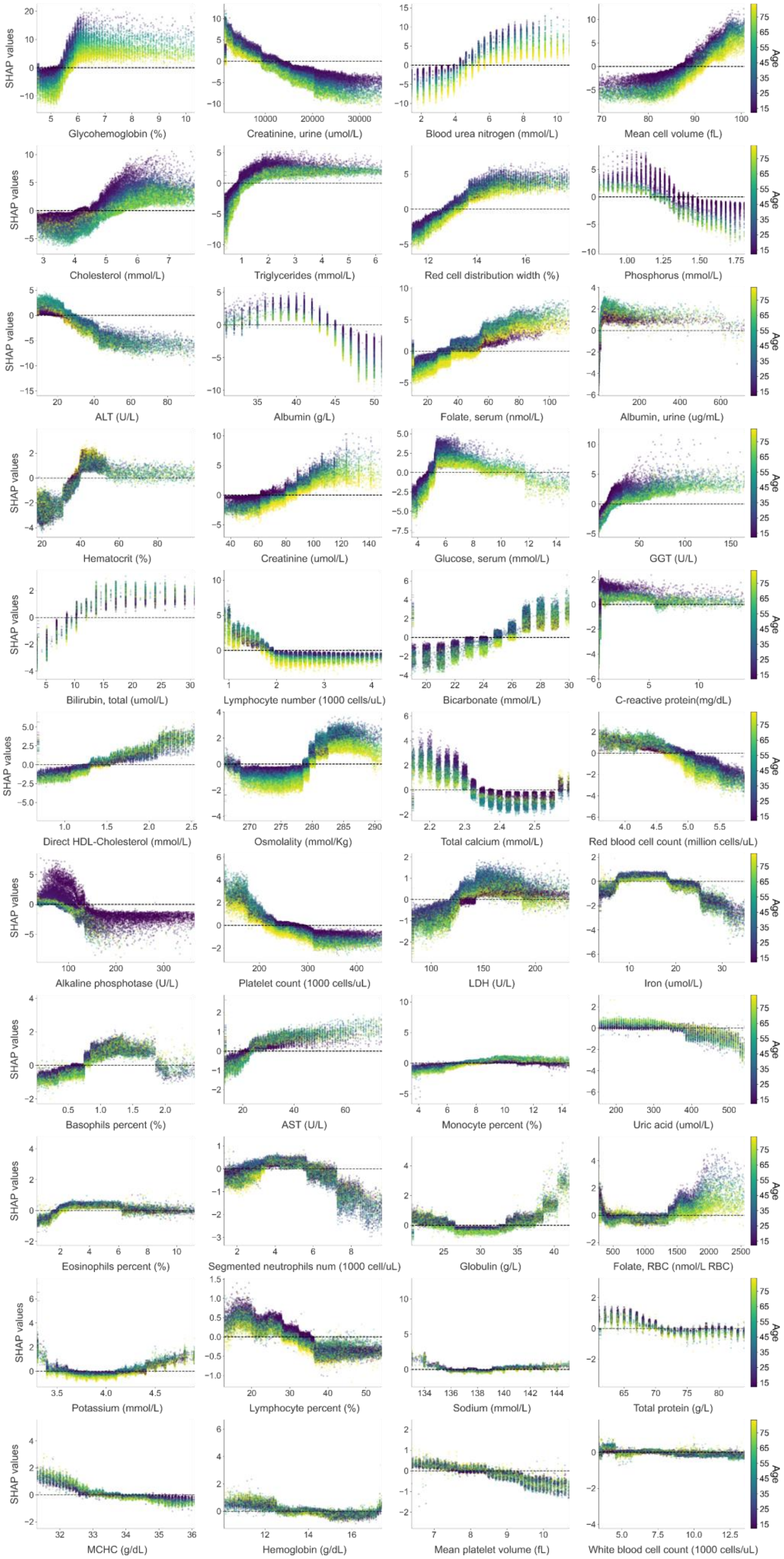

Fig. S10: Partial Dependence Plots of contextualized SHAP values.

(A) Contextualized SHAP values as a function of variable values. Each dot represents an individual. The color indicates the corresponding chronological age (scale on the right). X-axis corresponds to the real value of the variable, while the y-axis corresponds to the SHAP value given to this individual for this variable. The dotted line corresponds to the SHAP value of 0, which means that when the individual displays a variable value for which the SHAP value is 0, the variable has no impact on the physiological age.

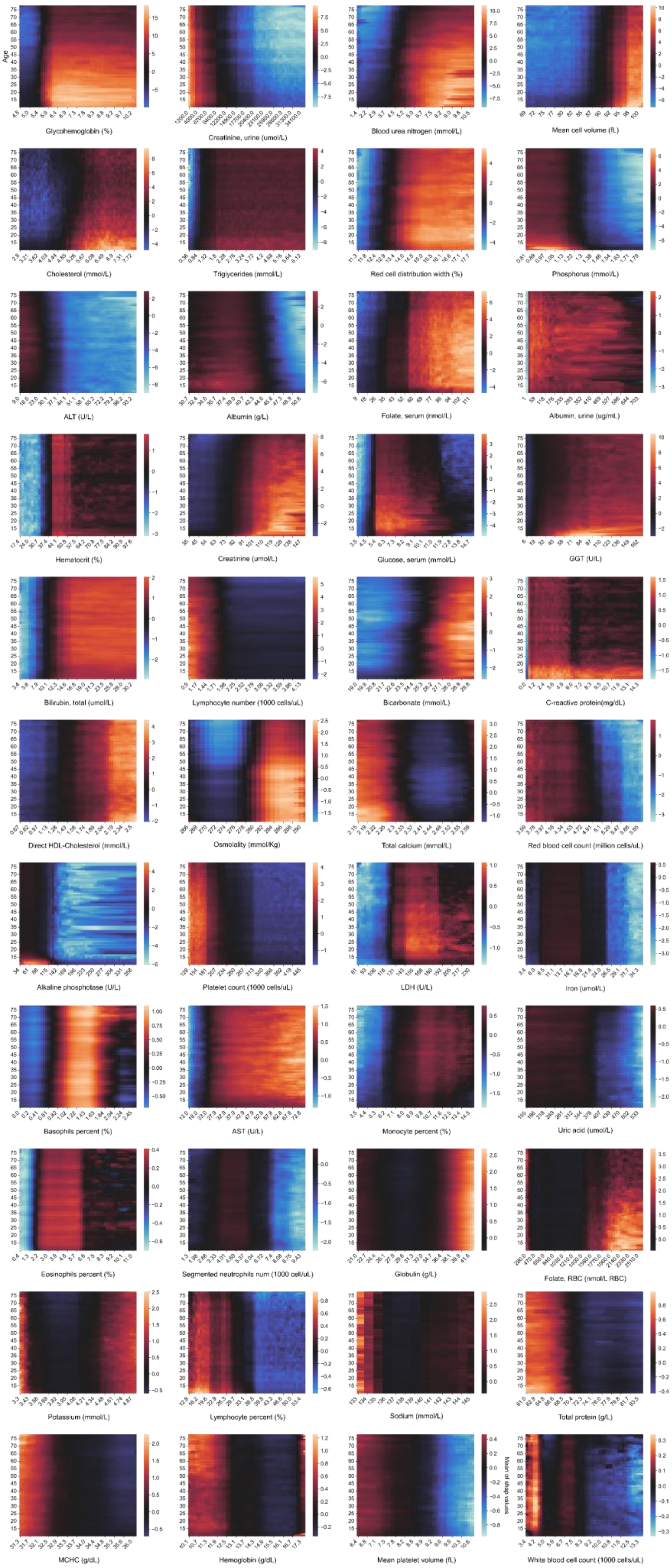

(B) Heatmap of contextualized SHAP values as a function of chronological age. The color of each pixel indicates the average SHAP value of a variable (x-axis) as a function of chronological age (y-axis).

Table S1: List of the 48 biological variables, by alphabetical order

SAS label	Excluded during feature selection
Albumin (g/L)	
Albumin, urine (ug/mL)	
Alkaline phosphotase (U/L)	
ALT (U/L)	
AST (U/L)	
Basophils number (1000 cells/uL)	
Basophils percent (%)	
Bicarbonate (mmol/L)	
Bilirubin, total (umol/L)	
Blood urea nitrogen (mmol/L)	
Cholesterol (mmol/L)	
C-reactive protein(mg/dL)	
Creatinine (umol/L)	
Creatinine, urine (umol/L)	
Direct HDL-Cholesterol (mmol/L)	
Eosinophils percent (%)	Yes
Folate, RBC (nmol/L RBC)	
Folate, serum (nmol/L)	
GGT (U/L)	
Globulin (g/L)	
Glucose, serum (mmol/L)	
Glycohemoglobin (%)	
Hematocrit (%)	
Hemoglobin (g/dL)	
Iron (umol/L)	Yes
LDH (U/L)	Yes
Lymphocyte number (1000 cells/uL)	
Lymphocyte percent (%)	
MCHC (g/dL)	
Mean cell hemoglobin (pg)	
Mean cell volume (fL)	
Mean platelet volume (fL)	Yes
Monocyte number (1000 cells/uL)	
Monocyte percent (%)	
Osmolality (mmol/Kg)	
Phosphorus (mmol/L)	
Platelet count (1000 cells/uL)	
Potassium (mmol/L)	
Red blood cell count (million cells/uL)	
Red cell distribution width (%)	
Segmented neutrophils num (1000 cell/uL)	
Segmented neutrophils percent (%)	
Sodium (mmol/L)	
Total calcium (mmol/L)	
Total protein (g/L)	
Triglycerides (mmol/L)	
Uric acid (umol/L)	
White blood cell count (1000 cells/uL)	

Table S2: List of hyperparameters used during model tuning.

The grid search is presented for each model together with the best hyperparameters found for each model.

<table>
<tr><th>Model</th><th>Grid search parameters</th><th>Best hyperparameters found</th></tr>
<tr><td>Elastic Net</td><td>l1_ratio: [0.01, 0.05, 0.1, 0.2, 0.3, 0.4, 0.5, 0.6, 0.7, 0.8, 0.9, 0.95, 0.99]
alpha: uniform (-4, -2, 0.5)</td><td>l1_ratio: 0.99
alpha: 0.0001</td></tr>
<tr><td>Random Forest</td><td>n_estimators: loguniform (100, 1000)
max_features: [auto, sqrt]
max_depth: randint (3,12)
min_samples_split: [2,5,10]
min_samples_leaf: [1,2,4]
bootstrap: [True, False]</td><td>n_estimators: 598
max_features: auto
max_depth: 11
min_samples_split: 5
min_samples_leaf: 2
bootstrap: True</td></tr>
<tr><td>Decision Tree</td><td>max_depth: int(2, 50)
min_samples_split: int(2, 12)
min_samples_leaf: int(2, 50)</td><td>max_depth: 28
min_samples_split: 6
min_samples_leaf: 24</td></tr>
<tr><td>Multilayer Perceptron</td><td>n_layers: [2,3,4] with hidden_layer_sizes [16,32,64,128,256]
activation: [relu, identity]
beta_1: [0.01, 0.05, 0.1, 0.2, 0.3, 0.4, 0.5, 0.6, 0.7, 0.8, 0.9, 0.95, 0.99]
beta_2: [0.01, 0.05, 0.1, 0.2, 0.3, 0.4, 0.5, 0.6, 0.7, 0.8, 0.9, 0.95, 0.99]
alpha: uniform (-4, -1, 0.5)</td><td>n_layers: 2 with hidden_layer_sizes (16,64,32,64)
activation: relu
beta_1: 0.1
beta_2: 0.4
alpha: 0.003</td></tr>
<tr><td>XGBoost Model</td><td rowspan="2">max_depth: [3,4]
subsample: uniform(0.2, 0.8, 0.05)
colsample_bytree: uniform(0.2, 1.0, 0.05)
colsample_bylevel: uniform(0.2, 1.0, 0.05)
learning_rate: 10^(uniform(-4.0, -1.0, 0.5))</td><td>max_depth: 3
subsample: 0.7
colsample_bytree: 0.85
colsample_bylevel: 0.9
learning_rate: 0.1</td></tr>
<tr><td>XGBoost Model with custom loss</td><td>max_depth: 3
subsample: 0.8
colsample_bytree: 1.0
colsample_bylevel: 0.5
learning_rate: 0.01</td></tr>
</table>

Table S3: Details of the validation variables

**Socio-demographic variables**	**Gender** (RIAGNDR, Male - Female)<br>**Ethnicity** (RIDRETH1, Non-Hispanic white - Mexican American - Non-Hispanic black - Other)<br>**Family income** : Annual Family Income (INDFMINC and INDFMIN2) divided into quartile<br>**Poverty Index Ratio** (INDFMPIR) divided into Poverty (<1) and No poverty (≥1)
**Medical variables**	**Body Mass Index** : BMXMI divided into Obesity (≥ 30), Overweight (≥25 and <30), **Normal weight**: (≥18.5 and <25) and Underweight (<18.5)<br>**Tobacco exposure** : LBXCOT (Cotinine) divided into No exposure (≤13) and Exposure (>13)<br>**Sedentarity**: combination of PAD020, PAQ635 (Walked or bicycled over past 30 days, Yes/No), PAQ180 (Avg level of physical activity each day, from no activity to high activity) and PAD320, PAQ620 (Moderate activity over past 30 days, Yes/No). Active defined as at least one activity among the previous ones<br>**AAC24 score:** DXXAAC24 (AAC Total 24 Score) recoded into Low score (0-1), Med score (2-5), and High score (6+)<br><br>**Pathologies**<br>Liver diseases: MCQ160L, MCQ500 (Ever told you had any liver condition)<br>Coronary Heart Diseases: presence of a condition among MCQ160C (Ever told you had coronary heart disease), MCQ160F (Ever told you had a stroke), MCQ160B (Ever told had congestive heart failure), MCQ160D (Ever told you had angina/angina pectoris) and MCQ160E (Ever told you had heart attack)<br>Diabetes: presence of a condition among DIQ010 (Doctor told you have diabetes) and DIQ160 (Ever told you have prediabetes)<br>Thyroid diseases: presence of a condition among MCQ160H (Ever told you had a goiter), MCQ160M (Ever told you had a thyroid problem) and MCQ160I (Ever told you had thyroid disease)<br>Arthritis: presence of a condition among MCQ160N (Doctor ever told you that you had gout?), MCQ160A (Doctor ever said you had arthritis) and ARQ125E (Ever told had Ankylosing Spondylitis)<br>Cancer: MCQ220 (Ever told you had cancer or malignancy)<br>Kidney diseases: presence of a condition among KIQ020, KIQ022 (Ever told you had weak/failing kidneys) and OHQ144 (Have kidney disease w/ renal dialysis?)<br>Bronchitis: presence of a condition among MCQ160K (Ever told you had chronic bronchitis), MCQ160o (Ever told you had COPD?) and MCQ010 (Ever been told you have asthma)<br>Auto-immune digestive disease: presence of a condition among ARQ125C (Ever told you had Ulcerative Colitis) and ARQ125D (Ever told you had Crohns Disease)<br>Digestive ulcer: MCQ200 (Ever told had stomach/duod/peptic ulcer)<br>Eye disease: presence of a condition among VIQ090 (Ever told had glaucoma) and VIQ310 (Told had macular degeneration)<br>Dermatologic disease: presence of a condition among DEQ053, MCQ070 (Ever told had Psoriasis?) and AGQ180 (Doctor told have eczema)
